# Supplementary material for: Radical SAM-dependent ether crosslink in daropeptide biosynthesis
Source: Nat Commun. 2022 Apr 29;13:2361. doi: 10.1038/s41467-022-30084-2 (PMC9055067; doi:10.1038/s41467-022-30084-2)
Supplement: Supplementary file 1 — Supplementary Information [file 41467_2022_30084_MOESM1_ESM.pdf]

Supplementary Information

Supplementary Methods

General experimental procedures. HPLC was performed using a Thermo Scientific Dionex UltiMate 3000 HPLC system equipped with a diode array detector (DAD). LC-HRMS analysis was carried out on a Q-Exactive^{TM} Focus Hybrid Quadrupole Orbitrap Mass Spectrometer coupled to a Thermo Scientific Dionex UltiMate 3000 UHPLC system with a multiple wavelength detector. NMR spectra were recorded on a Bruker Avance III HD 800 MHz NMR spectrometer in dimethyl sulfoxide-d_6. Polymerase chain reaction (PCR) was performed on a Bio-Rad T100^{TM} Thermal Cycler. The UV-vis spectrum was recorded on a Lamda750S spectrometer (PerkinElmer). Electroporation was carried out using mode Ec2 (2.5 kV) on a Bio-Rad (USA) MicroPulser Electroporator.

Chemicals and biochemicals. All chemicals and biochemicals were obtained from commercial sources and used without further purification unless otherwise specified. ACS/HPLC certified MeCN and MeOH were purchased from J&K Scientific (Beijing, China). Watsons water (distilled) was used as the LC and LC-MS analysis. Antibiotics (kanamycin for pRSFDuet and pET-28a(+) plasmids, ampicillin for pETDuet plasmid) were provided by Sangon Biotech (Shanghai, China). Phanta® Max Super-Fidelity DNA Polymerase was purchased from Vazyme Biotech (Nanjing, China). KOD One^{TM} PCR Master Mix was ordered from TOYOBO Biotech (Japan). Restriction enzymes were purchased from Takara Biotech (Dalian, China) or New England Biolabs (NEB, USA). Ni NTA beads were purchased from Smart lifesciences (Changzhou, China). Regular chemical reagents were ordered from Sinopharm Co. Ltd (Beijing, China). Culture media such as tryptone and yeast extract were brought from the Oxoid Inc. (Thermo Fisher Scientific, USA). Trypsin protease (≥7,500 BAEE units/mg solid) was purchased from Sigma-Aldrich (USA). *S*-adenosyl-L-methionine (SAM) was supplied by Macklin Inc. (Shanghai, China). $H_2{}^{18}O$ containing at least 98 atom % O-18 was purchased from Meryer (Shanghai) Chemical Technology Co., Ltd. Isopropyl β-D-thiogalactopyranoside (IPTG) was acquired from Sangon Biotech. Molecular biology kits including DNA purification kit, plasmid mini preparation kit, ClonExpress II one step cloning kit, ClonExpress MultiS One Step Cloning Kit, Mut Express II fast mutagenesis kit, and Bradford assay kit were brought from CWbio Co. Ltd (Beijing, China), TIANGEN Biotech (Beijing, China), and Vazyme Biotech. Chemically-competent *E. coli* cells comprising DH5α, BL21(DE3), and BL21 Star (DE3) were purchased from Vazyme Biotech. For desalting of proteins or fusion precursor peptides, PD-10 Minitrap G-25 columns (GE Healthcare, USA) were used. Synthetic genes inserted into expression vectors were obtained from Genewiz Co. Ltd (Suzhou, China). The constructed plasmids were verified by Sanger sequencing analysis offered by Genewiz Co. Ltd and Sangon Biotech. All oligonucleotide primers were purchased from Genewiz Co. Ltd and Sangon Biotech. Solid-phase peptide synthesis of $DarA_{pk}$ was offered by Sangon Biotech.

Culture media. All bacterial strains used in this study were grown in lysogeny broth (LB) medium at 37 °C and maintained in glycerol stocks (20% v/v) at − 80 °C. The LB medium was prepared with 10 g/L tryptone, 5 g/L yeast extract, and 5 g/L NaCl, and supplemented with 50 mg/L kanamycin or/and 100 mg/L ampicillin

when required for the purpose of selection. The terrific broth (TB) medium was made as follows: add 900 mL of water to 24 g yeast extract, 20 g tryptone, and 4 mL glycerol. Prepare 100 mL phosphate buffer (0.17 M KH_2PO_4, 0.72 M K_2HPO_4). Sterilize the respective solutions by autoclaving separately and allow the solution to cool to about 60 °C, prior to mixing them vigorously.

Plasmid construction. Coexpression of precursor peptide and the modifying rSAM enzymes were performed in a way similar to a recent study.[4] Synthetic $darA_{pk}$ and $darE_{pk}$ genes from *Photorhabdus khanii* HGB1456 were inserted into the first multiple cloning site (MCS1) included with a NHis tag and the second multiple cloning site (MCS2), respectively, at the *Bam*HI/*Xho*I restriction sites of the pRSFDuet based vector, thus creating plasmid *pRSFDuet-darAE*$_{pk}$. Similarly, the genes *darBCD*$_{pk}$ from the gene cluster were synthesized and then assembled to the pETDuet vector at the *Nco*I/*Xho*I restriction sites, constructing plasmid *pRSFDuet-darBCD*$_{pk}$. Note that all the intergenic regions were removed and the genes *darABCDE*$_{pk}$ were expressed streamlined under the control of T7-lac promoter.

Mutagenesis of the active-site [4Fe-4S] cluster was carried out on the constructed *pRSFDuet-darAE*$_{pk}$ using the Mut Express® II fast mutagenesis kit. One cysteine to alanine mutation (C83A) was made in a single mutagenesis step for the conserved N-terminal Fe-S cluster (247T>G, 248G>C) following the manufacturer's instructions. Single colonies were selected for Sanger sequencing and the correct $DarA_{pk}$/$DarE_{pk}$-C83A mutant was thus successfully produced.

Plasmid *pET28a-darA*$_{pk}$ carries the gene $darA_{pk}$ alone, which was amplified from the constructed *pRSFDuet-darAE*$_{pk}$. as template and then cloned into the pET-28a(+) based vector at *Bam*HI/ *Hin*dIII restriction sites via homologous recombination implemented by ClonExpress II kit.

After exhaustive attempts, we achieved soluble expression of RS enzyme $DarE_{pk}$ through co-expressing the modified enzyme $DarE_{pk}$ with the substrate $DarA_{pk}$. The purified PCR fragment $DarE_{pk}$ was assembled with a NHis tag into the MCS1 of pRSFDuet-1, which had been linearized using the *Bam*HI/*Hin*dIII restriction enzymes. On the other hand, fragment $DarA_{pk}$ lacking his-tag was inserted into MCS2 at *Nde*I/*Xho*I cloning sites though One-step assembly method, therefore generating the constructed plasmid *pRSFDuet-darEA*$_{pk}$.

In a similar manner, synthetic $darA_{yi}$ and $darE_{yi}$ genes from *Yersinia intermedia* were inserted into the MCS1 included with a NHis tag and the MCS2, respectively, at the *Bam*HI/*Xho*I cloning sites of the pRSFDuet based vector, consequently engendering plasmid *pRSFDuet-darAE*$_{yi}$.

Plasmid *pRSFDuet-darAE*$_{pa}$ carried synthetic DNA sequences of $darA_{pa}$ and $darE_{pa}$ from *Photorhabdus asymbiotica*, which were amplified and assembled to the *Bam*HI/*Xho*I l linearized pRSFDuet vector.

All these constructed plasmids were verified by Sanger sequencing. The constructed plasmids were subsequently transformed into chemically-competent *E. coli* BL21(DE3) or BL21 Star (DE3) cells by heat shock or electrocompetent cells for expression (vide infra), see Supplementary Table 3. The primers used for this study are listed in Supplementary Table 4.

Expression of full *dar* cluster. 1 µL of constructed *pETDuet-darBCD*$_{pk}$ harboring *darBCD*$_{pk}$ was added to 70 µL of *E. coli* BL21(DE3): $darAE_{pk}$ electrocompetent cells, which were transformed in a 2 mm electroporation cuvette. The transformed cells were then grown overnight at 37 °C on LB agar supplemented with 50 µg/mL kanamycin and 100 µg/mL ampicillin. The resulting double transformants carrying the *dar* cluster (*darABCDE*$_{pk}$)

were picked up and maintained with appropriate antibiotics for co-expression. 10 mL of freshly cultured cells were used to inoculate 1 L of TB medium, which was cultivated at 37 °C/200 rpm, until reaching an OD_{600} of 0.6–0.8. At that point, the two plasmids (*pRSFDuet-darAE$_{pk}$* and *pETDuet-darBCD$_{pk}$*) were induced with 0.2 mM IPTG. After the induction, the cells were incubated at 18 °C, with 120 rpm shaking for 24 h before sample treatment. For protein purification, the whole process was identical to that of co-expression of $DarAE_{pk}$ and the resulting peptide was further subjected to the LC-HRMS analysis (method A).

Sample preparation and detection of mature darobactin. The mature peptide darobactin was analyzed by the LC-HRMS approach. From the co-expression culture of $DarAE_{pk}$, 20 mL aliquot was taken and centrifuged at 4,500 rpm to separate the medium and the bacterial cell. The medium was lyophilized, 5 mL methanol was added, the mixture was sonicated for 30 min, and centrifuged at 14,000 rpm for 5min. The methanol was discarded, and the pellet was resuspended in 2 mL deionized water. After a final centrifugation for 5 min, the sample of culture medium was ready to be injected to LC-HRMS analysis. To prepare the sample from the cell pellet, 1 mL of methanol was added prior to sonication for 30 min. Next, 1 mL of deionized water was added, and the further sonication was continued for another 30 min. Thereafter, the solution was centrifuged to pelletize the insoluble part and the supernatant was sent for the LC-HRMS analysis. Both of the analytic conditions corresponded to the method A mentioned above.

Heterologous expression of $DarE_{pk}$. BL21 Star (DE3) cells harboring *pRSFDuet-darEA$_{pk}$* were obtained by electroporation and plated on LB agar plates containing 50 μg/mL kanamycin at 37 °C. After 20 h, single colonies were inoculated with 100 mL of LB containing the same concentration of antibiotics and grown at 37 °C for overnight. Saturated pre-cultures were inoculated into 1.5 L TB medium in a 1% (v/v) ratio and grown at 37 °C until reaching OD_{600} 1.5–2.0. For protein expression, cultures was induced with 0.2 mM IPTG and supplemented with 0.1 mM ferrous ammonium sulfate [$Fe(NH_4)_2(SO_4)_2$] and 0.1 mM L-cystine after cooling on ice for 30 min, and subsequently shaken at 37 °C, 150 rpm for 16-20 h before harvest. The cultures were harvested by centrifugation at 4,500 rpm for 10 min, washed with lysis buffer (10 mM imidazole, 20 mM MOPS, 200 mM NaCl, pH = 8.0), then flash-frozen and stored at –80 °C until use.

Protein purification was carried out in an anaerobic chamber under a strictly anaerobic condition (with less than 2 ppm of oxygen). All buffers used for purification were degassed and placed in the anaerobic chamber 24 h in advance. Cells were re-suspended in lysis buffer supplemented with 2 mg/ml lysozyme and protease inhibitor cocktail at 1:100 (v/v) dilution, and then incubated on ice for 1h. The culture was lysed via sonication according to the following procedure for 40 min on ice: 3 s sonication, 20 s interval with 35% power. Lysate was clarified by centrifugation at 13,000 rpm for 40 min at 4 °C and the supernatant decanted. Afterwards, Ni-NTA-based purification was performed in an anaerobic glove box as well. The supernatant was added to lysis-buffer I pre-equilibrated Ni-NTA resin and flowed though for three times. The column was washed with 10 column volumes of lysis buffer I followed by 10 column volumes of wash buffer (50 mM imidazole, 20 mM MOPS, 200 mM NaCl, pH = 8.0). $DarE_{pk}$ was eluted from Ni-NTA resin with elution buffer (500 mM imidazole, 20 mM MOPS, 200 mM NaCl, pH = 8.0) until resin recovery wathet. All fractions were analyzed by 10% SDS-PAGE gel and fractions containing $DarE_{pk}$ were pooled and concentrated by a 30,000 Da molecular weight cut-off Amicon Ultra centrifugal filter. The concentrated protein was then exchanged into desalting buffer I (50

mM MOPS, 25 mM NaCl, 10 mM dithiothreitol, 2.5% glycerol) using a PD-10 desalting column per manufacturer's protocol. Protein concentration was quantified using Bradford assay kit based on bovine serum albumin (BSA) as a standard.

In vitro chemical reconstitution of DarE$_{pk}$. Reconstitution of DarE$_{pk}$ was performed according to a standard procedure.[5] Briefly, fresh solutions of dithiothreitol (DTT, 1 M), sodium sulfide ($Na_2S\cdot9H_2O$, 50 mM), and ferrous iron ($(NH_4)_2Fe(SO_4)_2$, 500 mM) were prepared under strictly anaerobic condition. The concentration of DarE$_{pk}$ was adjusted to 100 μM and maintained at 4 °C in an anaerobic chamber. Subsequently, freshly prepared DTT was added to protein solution to a final concentration of 10 mM and $(NH_4)_2Fe(SO_4)_2$ was carefully added to a final concentration of 1 mM. After a 10 min incubation on ice, the mixture was supplemented with $Na_2S\cdot9H_2O$ repeatedly for three times until a final concentration reaching 1 mM, each time incubated on ice for 20 min. The reconstitution reaction was further proceeded at 4 °C for 16-18 h, after which, excess iron and sulfur were removed from blackish reaction mixture via PD-10 column as described above.

***S*-adenosyl-L-methionine (SAM) cleavage assay for DarE$_{pk}$.** To perform SAM cleavage assay, ~10 μM DarE$_{pk}$ was incubated with 2 mM SAM and 5 mM sodium dithionite in 20 mM Tris buffer (pH = 8.0). Reaction volumes were typically 100 μL and were maintained at r.t. in glove box for 2 h prior to quenching. The reactions were quenched by boiling at 95 °C for 10 min. After removal of the protein precipitates by centrifugation, the supernatant was sent for the LC-HRMS analysis.

LC-HRMS (method B) was performed in positive ion mode using a C18 column (Accucore XL C18, Thermo Scientific, 150 × 2.1 mm, 4 μm). The column was equilibrated with 98% solvent A (0.1% formic acid in H_2O) and 2% solvent B (MeCN), and developed at a flow rate of 0.3 mL/min: 0–1.5 min, 2% B; 1.5–3.5 min, a linear gradient to 95%B; 3.5–5 min, 95%B.

Heterologous expression of DarA$_{pk}$ and purification. In order to heterologously express DarA$_{pk}$, plasmids *pET28a-darA$_{pk}$* were transformed into chemically-competent BL21 (DE3) cells by heat shock. The transformants were obtained and protein expression were performed as previously described. For protein purification, the whole process was analogous to that of co-expression of DarAE$_{pk}$. The expected peptides were stored in −80 °C upon further LC-HRMS analysis (method A).

Solid phase peptide synthesis (SPPS). Peptides were synthesized using standard fluorenylmethyloxy-carbonyl (Fmoc) based solid phase peptide synthesis (SPPS) techniques using a Rainin PS3 peptide synthesizer. Preloaded resin (either Wang or 2-chlorotrityl; 0.1 mmol) was first swollen in dimethylformamide (DMF). Fmoc-amino acids (0.4 mmol, 4 equiv) were coupled using O-(1H-6-chloro-benzotriazol-1-yl)-N,N,N0,N0-tetramethyluronium hexafluorophosphate (HCTU, 165 mg, 0.4 mmol, 4 equiv) as coupling reagent and 0.4 M N-methyl morpholine (NMM) as activating reagent (45 min). Fmoc deprotection was performed with piperidine. After completion of the coupling of the final amino acid, the Fmoc group was removed to generate the free amino group.

Peptides were cleaved from the resin by adding a solution of trifluoroacetic acid (TFA) (5 mL), triisopropylsilane (TIPS) (100 μL), and H_2O (100 μL) to the resin (0.1 mmol), and stirring the solution for 2 h

at room temperature. The solution was concentrated by purging with a nitrogen stream and peptides were precipitated with cold diethyl ether. The crude peptides were dissolved in 0.1% aqueous TFA, lyophilized, and purified by preparative RP-HPLC. HPLC fractions containing products as confirmed by mass spectrometry were collected and lyophilized.

Enrichment and isolation of the modified tryptic fragment DarA$_{yi(55\text{-}66)}$-1. Large-scale heterologous co-expression of DarAE$_{yi}$ to generate enough product for NMR analysis was carried out on about 60 L scale according to the previous procedure. The full-length peptides were purified via Ni-affinity column, then desalted with semi-preparative HPLC, and dried by lyophilization, affording over 200 mg dried material. The obtained peptides were at batches resuspended in 20 mM Tris buffer (pH = 8.0) and digested with trypsin protease. The reaction was typically carried out on a 500 μL with 10 mg peptide, and trypsin was added to a final concentration of 100:1 substrate to enzyme. Reactions were allowed to incubate for about 6 hours at 37 °C.After the proteolytic digestion, the reaction mixtures were boiled and centrifugated. The pellets were resuspended in denaturing buffer I and centrifugated. The resulting supernatant was then subjected to analysis by HPLC equipped with a YMC-Triart C8 analytical column (4.6 × 250 mm, 5 μm). Next, repeated injections were performed on a semi-preparative YMC-Triart C8 column (10.0 × 250 mm, 5 μm). The desired tryptic fragment was eluted with a linear gradient of 5–25% MeCN (25 min) over 16 min at a flow rate of 3 mL/min. Fractions containing the expected product were pooled and lyophilized, yielding the final peptide (DarA$_{yi(55\text{-}66)}$-**1**, 2 mg).

Structural elucidation of the tryptic fragment DarA$_{yi(55\text{-}66)}$-1. On the basis of the doubly protonated ion at m/z 740.3335 $[M+2H]^{2+}$ in the HRMS spectrum, its molecular formular was determined to be $C_{68}H_{90}N_{18}O_{20}$ with 33 double bond equivalents (DBEs). The observation of a number of exchangeable amide NH protons (δ_H 5.6–8.4 ppm) from ^{1}H NMR assured its peptidic nature. Although a high degree of spectral overlap existed, twelve spin systems were assembled from the integrative analysis of 2D NMR data including COSY, HSQC, and NOESY spectra, Supplementary Figure 13-15. Briefly, among the deduced subunits were the amino acids N-terminal Ile, 2 × Ala, 2 × Ser, 2 × Trp, 1 × Arg, 1 × Thr, 1 × Asn, 1 × Phe, and C-terminal Glu. Notably, some abnormal resonances could be seen in the residues Trp4, Trp6, and Arg8. The aromatic regions of the ^{1}H NMR only exhibited four peaks associated with each Trp, and no signals could be attributed to the W^4-H_7 and W^6-H_6, indicative of quaternary carbons at the two aromatic positions. Consistent with this fact was the absent COSY correlations between H_7 and H_6 for W^4 or W^6. Likewise, the β positions of W^6 and R^8 were now occupied by the substituted methines. Importantly, a significant downfield shift of W^6-H_β at 5.99 ppm strongly proved it to be oxidized at C_β, a new methine at 75.19 ppm. The defined residues were step-wise connected via a combination of HMBC correlations from α-hydrogens to the adjacent amide carbonyls and the cross-peaks of $H_{\alpha,\beta}(i)$ to NH(i+1) in the NOESY spectrum to afford the linear sequence of DarA$_{yi(55\text{-}66)}$-1, which gave a congruent result for the *in silico* predicted peptide sequence. The so far delineated structure accounted for 31 out of the 33 total DBEs, necessitating two cyclization events to satisfy the final mass data. Digesting all the information above, we could anticipate the presence of a Trp6C_6-to-Arg8C_β crosslink through a new C(sp^2)–C(sp^3) bond and an ether linkage between C_β of W^6 and C_7 of W^4, although we were unable to obtain the conclusive correlations from the HMBC spectrum owing to the low sample quantity. Collectively, the tailing enzyme DarE alone installs two consecutive Trp6-Arg8 C–C and Trp4-Trp6 C–O–C crosslinks on its peptide

substrate, thereby generating a unique fused bicyclo modification. And at this point, we have completed the 2D structure of DarA$_{yi(55-66)}$-**1** as shown. This doubly cross-linked pattern was identical to that of reported darobactin, nevertheless there is little overall resemblance between the two 1D NMR spectra, largely due to the differently applied solvents (DMSO-d_6 vs. 94%H_2O:4%D_2O:2%formic acid-d_2).

As for the configurational discussion, the macrocycle crosslinks engender two stereogenic centers at the β-carbons of W^6 and R^8. The approximate *trans*-axial relationship between H_α and H_β of R^8 was determined by their large vicinal coupling (^3J = 9.2 Hz, Supplementary Figure 15). Based on a biosynthetic logic of the enzyme employed, the ribosomal synthesis of precursor DarA suggests that the Cα positions for the amino acid backbone should remain the stereochemical fidelity as L-configurations. Thereafter, the newly formed chiral center at R^8-C_β was assigned as *S*-configuration, which was further supported by the intense NOE correlation between H_β and NH of R^8 (Supplementary Figure 15). In a similar manner, the C_β of W^6 was accordingly determined to be *R*-configuration. On the other hand, taking into account the asymmetric nature of the *O*-indole ether bridge (W^4) and the indole cyclophane bridge (W^6), coupled with hindered rotation, it is conceivable that the substituted indoles would bring about the distinct types of planar chirality. To probe this hypothesis, we extensively analyzed the NOESY correlations within the macrocycle to assign the orientation of the modified indole rings. Further observations of dipolar correlations within the WNWSR macrocycle, in conjunction with the molecular modelling studies as shown in main text, clearly favor that both of the two cyclophanes indeed have planar chirality and should adopt $7S_p$ of W^4 and $6S_p$ of W^6 configurations, respectively, mainly based on the correlations of (1) W^4-H_2/-H_α/N^5-NH, W^4-H_4/-H_β, and W^6-H_6/-H_β (2) W^6-H_2/-H_α/W^4-H_1, W^6-H_4/-H_β, W^6-H_5/R^8-H_β/-NH, and W^6-H_7/R^8-H_α/-H_γ/-H_δ (Figures S15).

Recent years have witnessed a sharp increase in the use of quantum chemistry calculations in solving structure elucidation and validation problems, expedited by the remarkable advances in computer methodologies and hardware. Strikingly, the accuracy of the gauge-including atomic orbital (GIAO) method has been largely improved and is now around 2.0 ppm for ^{13}C NMR chemical shifts, which has made the density functional theory (DFT)-based structural elucidation quite effective.[6, 7] In this context, to verify the proposed structure, especially for the newly furnished macrocyclic core, we took intuitive to perform the ^{13}C NMR chemical shift calculations by DFT-GIAO approach. After a short screening of functionals, we turned our attention to the light level of theory [ωB97X-D/6-31G(d)// B3LYP/6-31G(d)]. In order to reduce computational cost, we truncated the system as shown in Supplementary Figure 16, maintaining that the omitted side residues do not significantly affect the calculated chemical shifts of the rigid macrocyclic moiety. Much to our satisfactory, after the linear scaling, this method displayed minor errors (R^2 = 0.999, CMAE = 0.94 ppm, CRMSD = 1.10 ppm, CMaxErr = 2.36 ppm, see Supplementary Figure 16), thus leaving little doubt that the structure of our model molecule of interest was indeed assigned correctly.

Computational details. The 3D structure of the model substructure DarA$_{yi(55-66)}$-**1** was firstly established according to 2D NOESY spectra and ^3J couplings. Conformational searches were run with Spartan 14 (Wavenfunction, Irvine, CA, USA, 2014) using the Monte Carlo algorithm and Merck molecular force field (MMFF94) with standard parameters and convergence criteria with a maximum of 10,000 steps. The MMFF minima, within an energy window of 5 kcal/mol, found by conformational search were analyzed using the

derived NOEs and ^3J couplings as postprocessor filter. The low-energy conformers corresponding perfectly to NOE and $^3J_{HH}$ filter were optimized with DFT method at B3LYP/6-31G(d) level in gas phase with the help of Gaussian 16 package, and were further checked by frequency calculation to ascertain no imaginary frequencies. GIAO ^{13}C NMR computation was implemented with the following components: (a) magnetic shielding: ωB97xD/6-31G(d) with the PCM for dimethyl sulfoxide; (b) scaling of isotropic magnetic shielding tensors according to the reported parameters to obtain chemical shifts. (c) The obtained values of chemical shifts in ppm are further linearly corrected with experimental chemical shifts. Such additional linear scaling is believed to better match the experimental values in more polar solvents, especially for dimethyl sulfoxide.

Supplementary Figure 1. Representative rSAM-dependent cyclic RiPPs. The rSAM-mediated RiPPs subfamilies via C–C bond formation include xenorceptide, ryptides (R-Y-crosslinked peptides), pyrroloquinoline quinone (PQQ), streptide, a tetrahydrobenzindole motif-containing peptide, and mycofactocin. Examples of cyclic RiPPs via C–S bond formation contain sactipeptides like streptosctin and ranthipeptides (radical non-a-carbon thioether peptides). Rotapeptides (radical oxygen-to-alpha-carbon-linked peptides) is the first number of ether-containing RiPPs family. Darobactin A holds an unprecedented scaffold bearing ether linkage and C–C bond double crosslinks, although the mechanism employed is not yet understood. The bonds forming the macrocycle are highlighted in cyan. Enzymes responsible for macrocyclization of peptides are shown in parentheses.

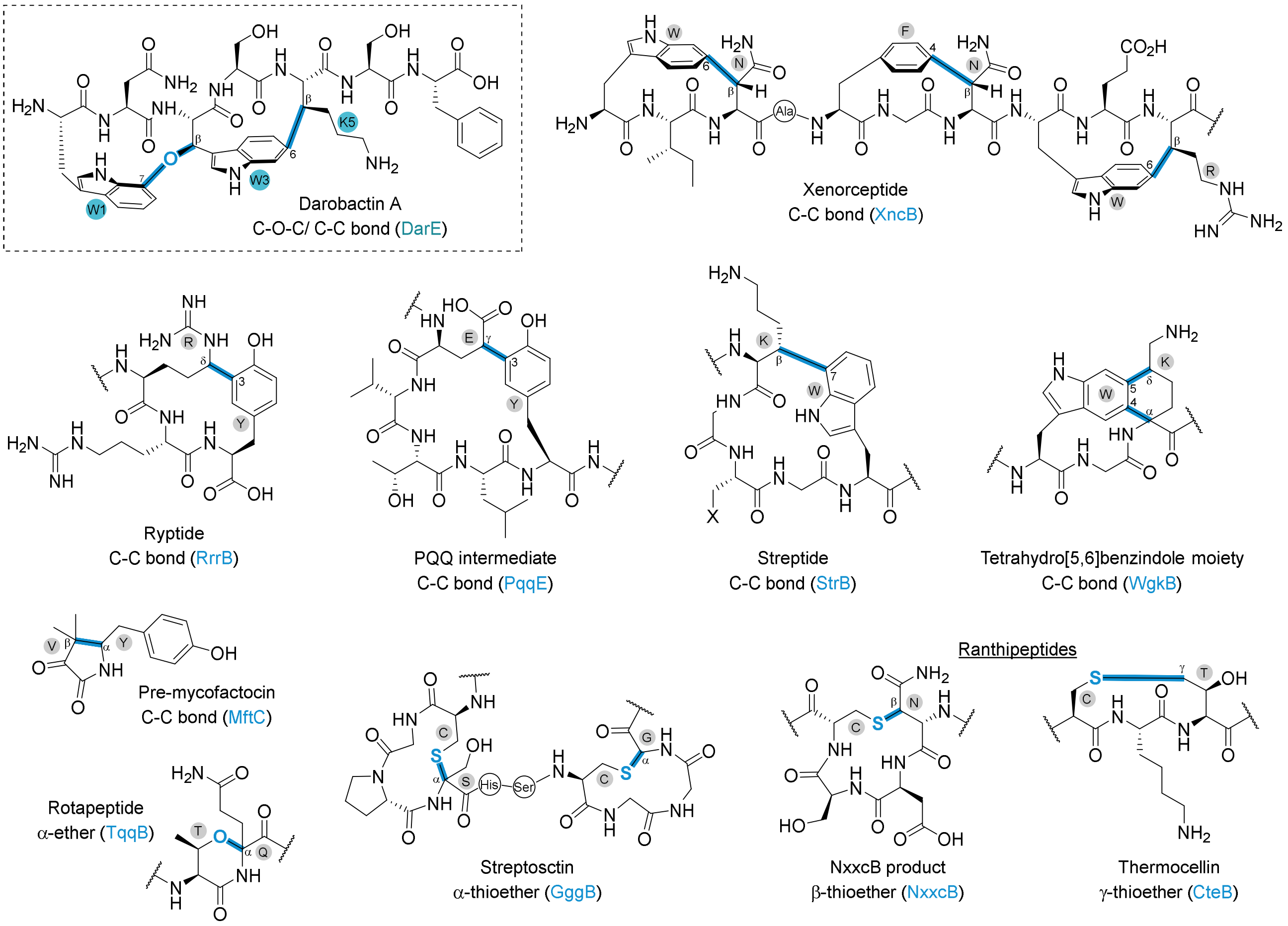

Supplementary Figure 2. Constructs used for heterologous expression of darobactin BGC in *E. coli* BL21(DE3). Each gene was expressed under the control of a separate T7 promoter, and all intergenic regions were removed.

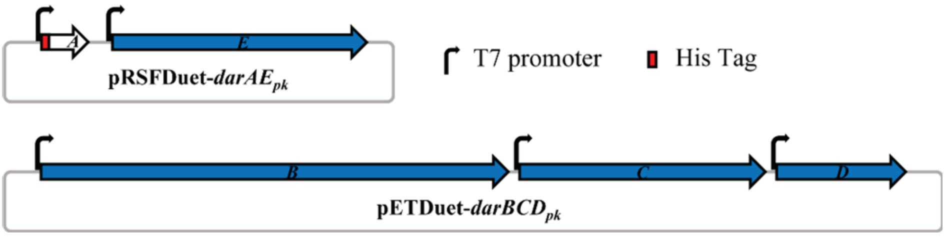

Supplementary Figure 3. HR-MS/MS analysis of DarA$_{pk(43-58)}$**-1**, showing (A) the shorthand representation and (B) MS/MS spectrum of DarA$_{pk(43-58)}$**-1**.

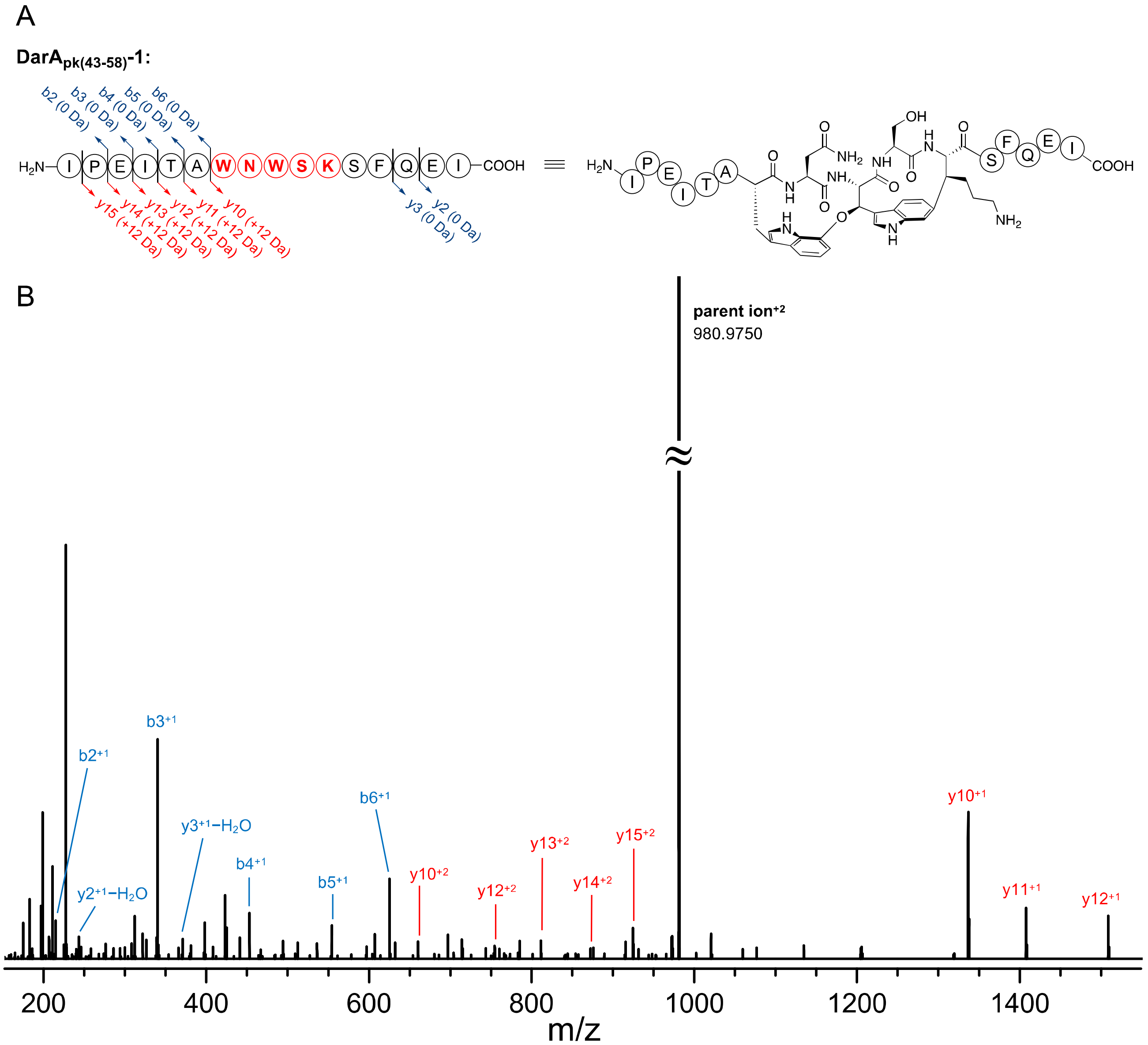

MS/MS data assignment of the key tryptic fragments DarA$_{pk(43-58)}$**-1**

Ion	Sequence	Obs.	Calc.	Error (ppm)	Shift (ΔDa)	Note
M+2H	IPEITAWNWSKSFQEI	980.9750 (z = 2)	980.9756 (z =2)	−0.31	11.9624	[O]−4×[H]
b2	IP	211.1434	211.1441	−3.32	-	
b3	IPE	340.1861	340.1867	−1.76	-	
b3-H_2O	IPE	322.1763	322.1761	0.62	-	
b4	IPEI	453.2715	453.2708	1.54	-	
b5	IPEIT	554.3196	554.3184	2.16	-	
b5-H_2O	IPEIT	536.3077	536.3079	−0.37	-	

b6	IPEITA	625.3550	625.3556	−0.96	-	
b6-H_2O	IPEITA	607.3421	607.3450	−4.77	-	
y2-H_2O	EI	243.1339	243.1339	0	-	
y3-H_2O	QEI	371.1914	371.1925	−2.96	-	
y10	WNWSKSFQEI	1336.5923	1336.5957	−2.54	11.9602	[O]−4×[H]
		668.7953 (z = 2)	668.8015 (z = 2)	−4.64	11.9512	
y11	AWNWSKSFQEI	1407.6328	1407.6328	0	11.9636	[O]−4×[H]
		704.3199 (z = 2)	704.3200 (z = 2)	−0.07	11.9634	
y12	TAWNWSKSFQEI	1508.6831	1508.6805	1.72	11.9662	[O]−4×[H]
		754.8375 (z = 2)	754.8439 (z =2)	−4.24	119508	
y13	ITAWNWSKSFQEI	811.3859 (z = 2)	811.3859 (z =2)	0	11.9636	[O]−4×[H]
y14	EITAWNWSKSFQEI	875.9110 (z = 2)	875.9072 (z =2)	2.17	11.9712	[O]−4×[H]
y15	PEITAWNWSKSFQEI	924.4369 (z = 2)	924.4336 (z =2)	1.79	11.9702	[O]−4×[H]

Supplementary Figure 4. LC-HRMS analysis of DarA$_{pk}$-**1** in vivo. The extracted ion chromatograms (EICs) at m/z = 1010.99 ± 0.1 correspond to the fully modified DarA$_{pk}$-**1** ([M + 8H]$^{8+}$). Coexpression of *darA$_{pk}$* with *darE$_{pk}$* (EIC trace i), or additional introduction of transporter genes *darBCD$_{pk}$* (EIC trace ii) did not show apparent difference in the yield and modification pattern of DarApk.

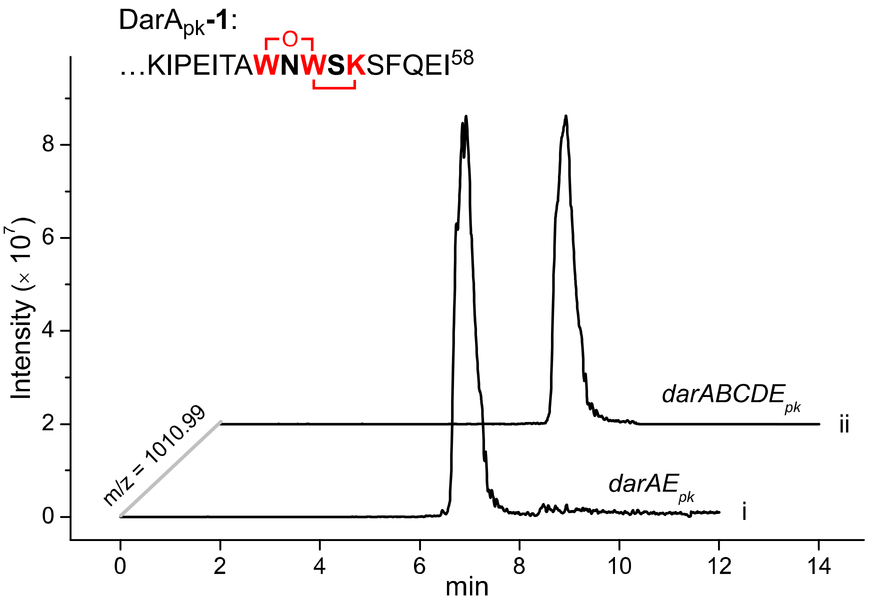

Supplementary Figure 5. Production of the mature darobactin in *E. coli*, showing the EIC (m/z = 483.71 ± 0.01) and the MS spectrum of darobactin.

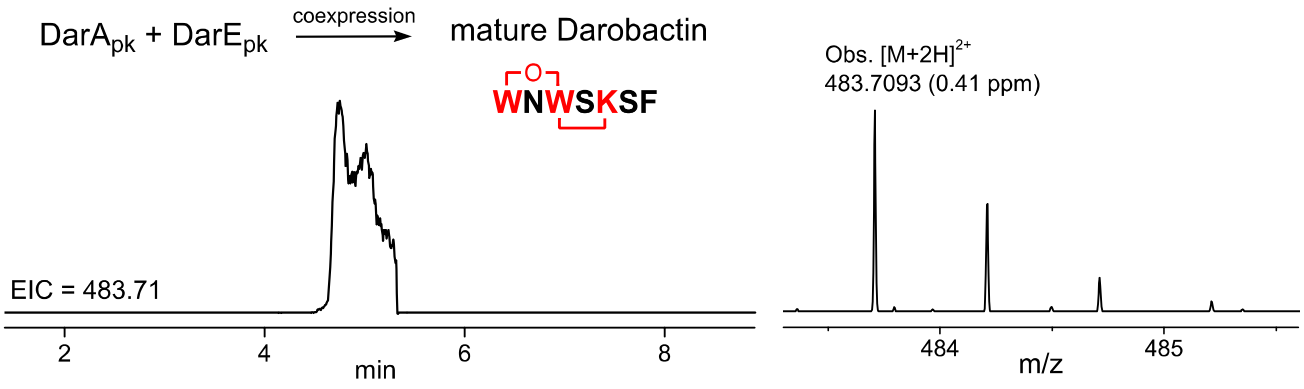

Supplementary Figure 6. In vitro proteolysis for darobactin maturation, showing the EIC at *m/z* = 483.71 ± 0.01 (corresponding to darobactin) of $DarA_{pk}$-1 incubated with $DarE_{pk}$ (blue trace) or the cell lysate *E. coli* BL21 (red trace) for 24h.

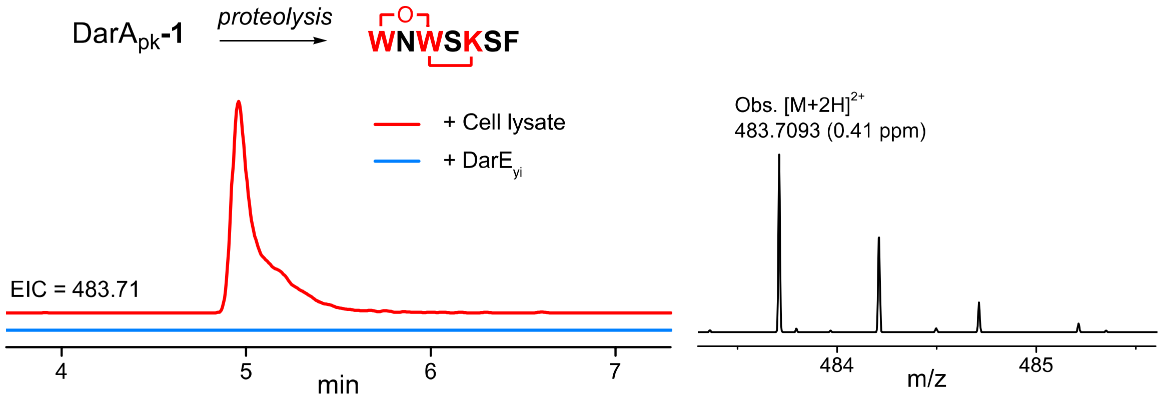

Supplementary Figure 7. In vitro analysis of DarE$_{pk}$. a). SDS-PAGE analysis of DarE$_{pk}$ purified by Ni-affinity chromatogram. b). UV–vis spectrum of the as-purified (black line) and the reconstituted (red dash) DarE$_{pk}$. c). LC-HRMS analysis of the DarE$_{pk}$-catalyzed dAdoH production. EIC at m/z = 252.11 ± 0.01 corresponds to dAdoH, the characteristic product of rSAM enzymes. The analyses were all performed more than three times independently, and the results are very similar.

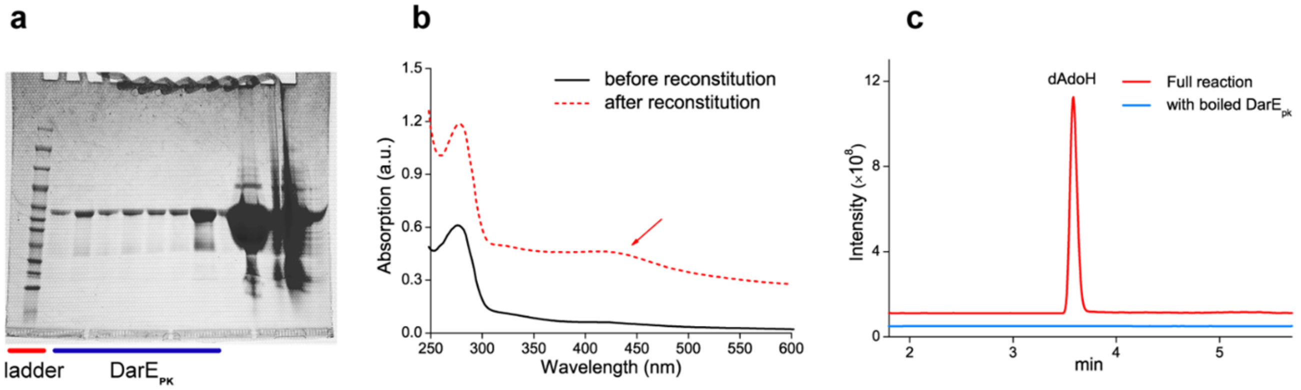

Supplementary Figure 8. HR-MS/MS analysis of DarA$_{pk(43-53)}$-**2**, showing (A) the shorthand representation and (B) MS/MS spectrum of DarA$_{pk(43-53)}$-**2**.

A

DarA$_{pk(43-53)}$:

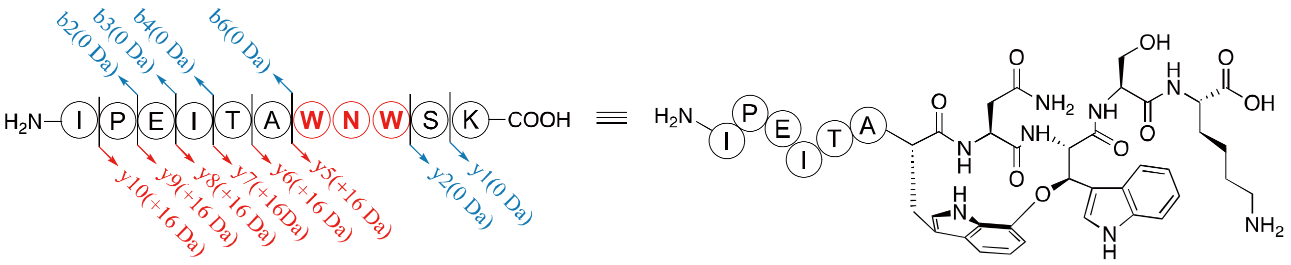

B

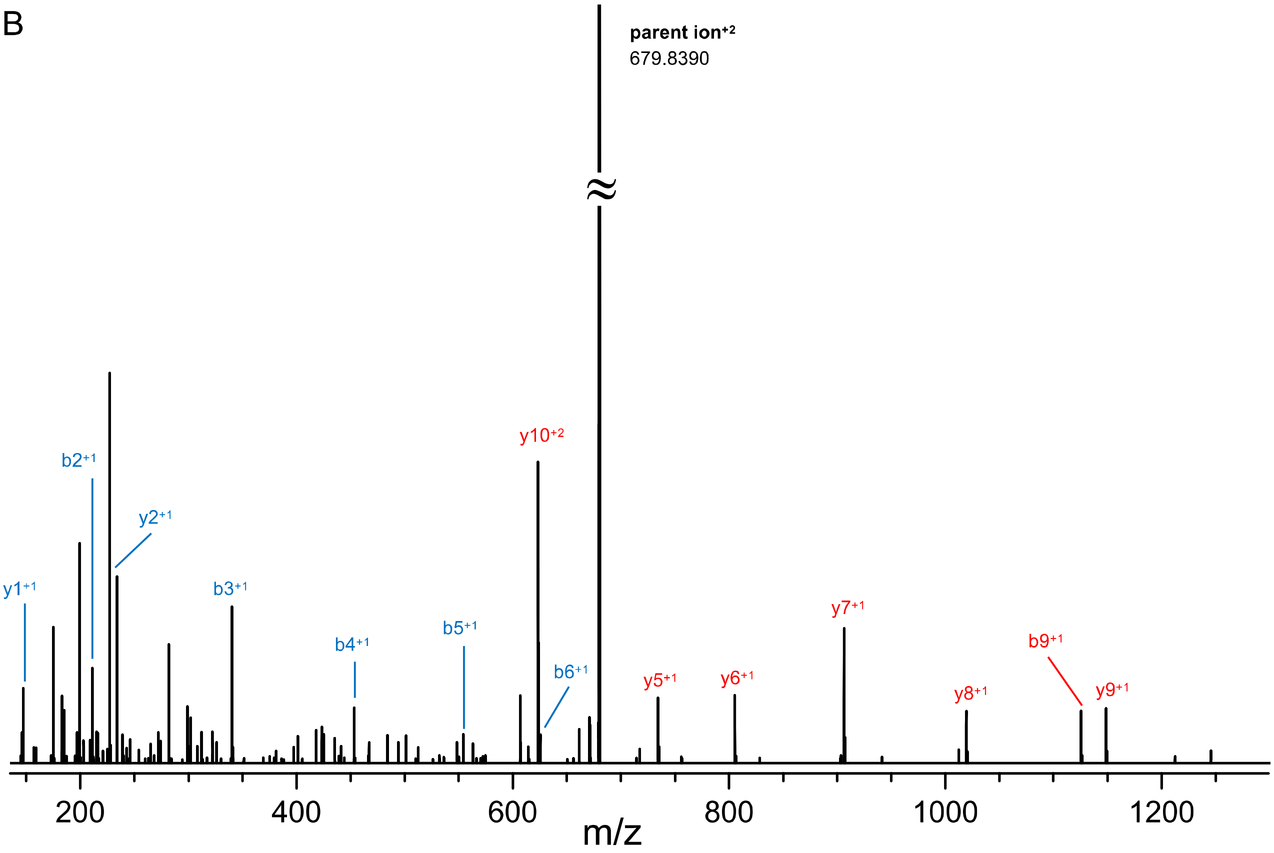

MS/MS data assignment of the key tryptic fragments DarA$_{pk(43-53)}$-**2**

Ion	Sequence	Obs.	Calc.(unmod.)	Error (ppm)	Shift (ΔDa)	Note
M+2H	IPEITAWNWSK	679.8390 (z = 2)	672.8406 (z =2)	−1.2	13.9760	[O]−2×[H]
b2	IP	211.1441	211.1441	0	-	
b3	IPE	340.1860	340.1867	−2.1	-	
b4	IPEI	453.2702	453.2708	−1.3	-	
b5	IPEIT	554.3204	554.3184	3.1	-	
b6	IPEITA	625.3563	625.3556	1.1	-	

b9	IPEITAWNW	1125.5359	1125.5364	−0.4	13.9788	[O]−2×[H]
y1	K	147.1128	147.1128	0	-	
y2	SK	234.1445	234.1448	−1.3	-	
y5	WNWSK	734.3237	734.3257	−2.7	13.9773	[O]−2×[H]
y6	AWNWSK	805.3595	805.3628	−4.1	13.9760	[O]−2×[H]
y7	TAWNWSK	906.4104	906.4104	0	13.9792	[O]−2×[H]
y8	ITAWNWSK	1019.4930	1019.4945	−1.5	13.9778	[O]−2×[H]
y9	EITAWNWSK	1148.5400	1148.5371	−2.5	13.9822	[O]−2×[H]
y10	PEITAWNWSK	1245.5726	1245.5899	−13.9	13.9620	[O]−2×[H]
		623.2984 (z = 2)	623.2986 (z =2)	−0.2	13.9790	

Supplementary Figure 9. LC-HRMS and HR-MS/MS analysis of the tryptic fragement of the $DarA_{pk}$ K53A mutant coexpressed with $DarE_{pk}$. (A) The extracted ion chromatograms (EICs) at m/z = 953.45 ± 0.1 corresponding to the +14 Da modification. (B) The shorthand representation structure and MS/MS spectrum of $DarA(K53A)_{pk(43-58)}$ after trypsin digestion.

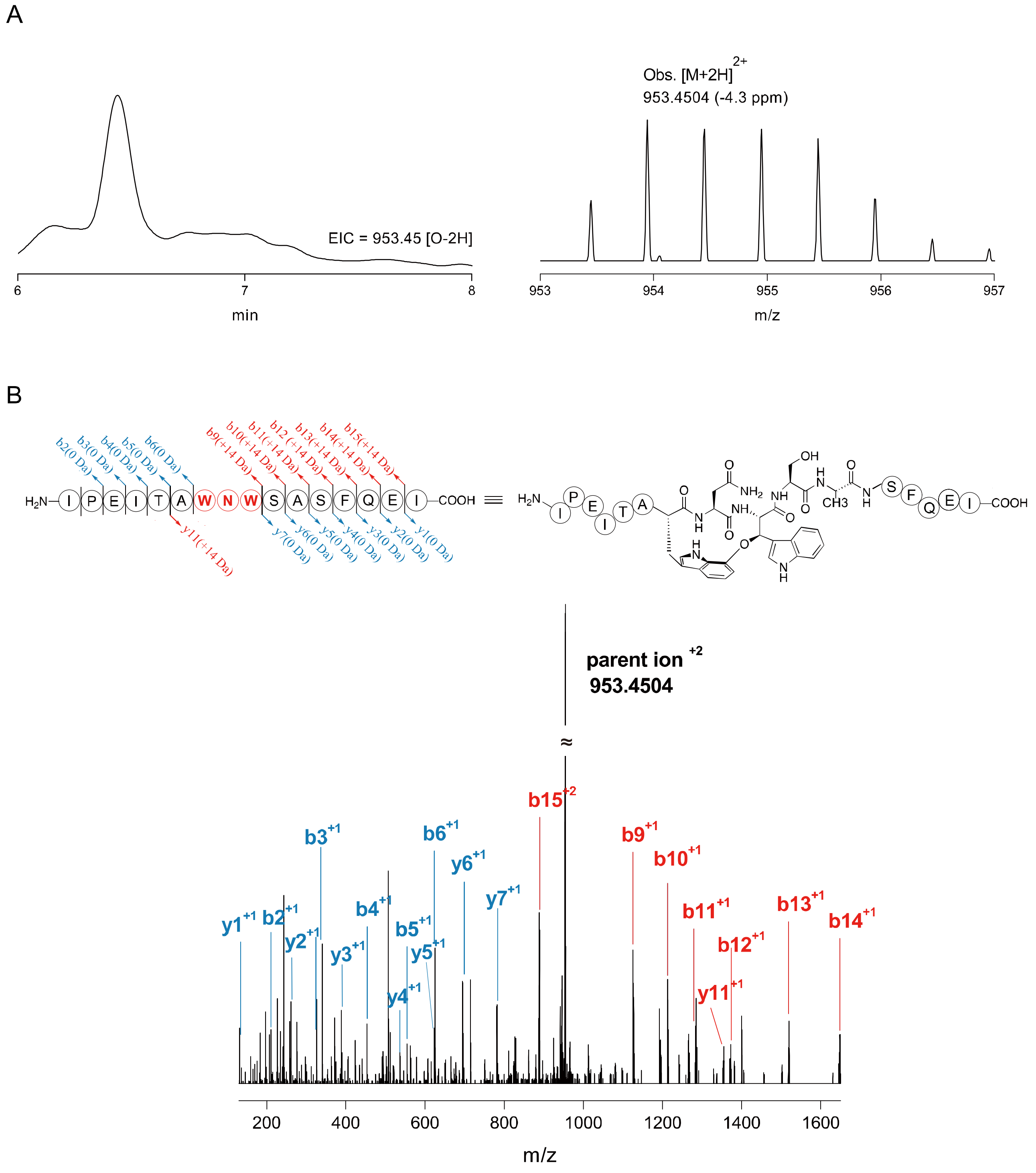

Ion	Sequence	Obs.	Calc.(unmod.)	Error (ppm)	Shift (ΔDa)	Note
M+2H	IPEITAWNWSASFQEI	953.4504 (z = 2)	953.4545 (z =2)	-4.3	13.9793	[O]−2×[H]
b2	IP	211.1429	211.1441	-5.68	-	
b3	IPE	340.1845	340.1867	-6.47	-	
b4	IPEI	453.2681	453.2708	-5.96	-	
b5	IPEIT	554.3166	554.3184	-3.25	-	
b6	IPEITA	625.3541	625.3556	-2.4	-	
b9	IPEITAWNW	1125.5313	1111.5571	-4.53	13.9742	[O]−2×[H]
b10	IPEITAWNWS	1212.5623	1198.5891	-5.03	13.9732	[O]−2×[H]
b11	IPEITAWNWSA	1283.5911	1269.6262	-11.2	13.9649	[O]−2×[H]
b12	IPEITAWNWSAS	1370.6323	1356.6583	-3.79	13.9740	[O]−2×[H]
b13	IPEITAWNWSASF	1517.6930	1503.7267	-8.57	13.9663	[O]−2×[H]
b14	IPEITAWNWSASFQ	1645.7410	1631.7853	-14.28	13.9557	[O]−2×[H]
b15	IPEITAWNWSASFQE	887.9034（z=2)	880.9176 (z=2)	-4.28	13.9716	[O]−2×[H]
y1	I	132.1013	132.1019	-4.54	-	
y2	EI	261.1429	261.1445	-6.13	-	
y3	QEI	389.2011	389.2031	-5.14	-	
y4	FQEI	536.2623	536.2715	17.16	-	
y5	SFQEI	623.2983	623.3035	-8.34	-	
y6	ASFQEI	694.3353	694.3406	-7.63	-	
y7	SASFQEI	781.3665	781.3727	-7.93	-	
y11	AWNWSASFQEI	1352.6111	1338.6113	15.16	13.9998	[O]−2×[H]

Supplementary Figure 10. HR-MS/MS analysis of DarA$_{pk(43-53)}$-**3**, showing (A) the shorthand representation and (B) MS/MS spectrum of DarA$_{pk(43-53)}$-**3**.

A

DarA$_{pk(43-53)}$:

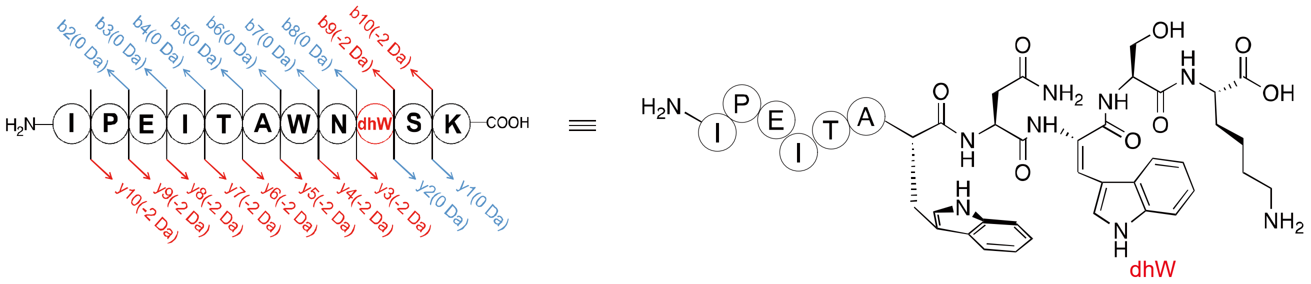

B

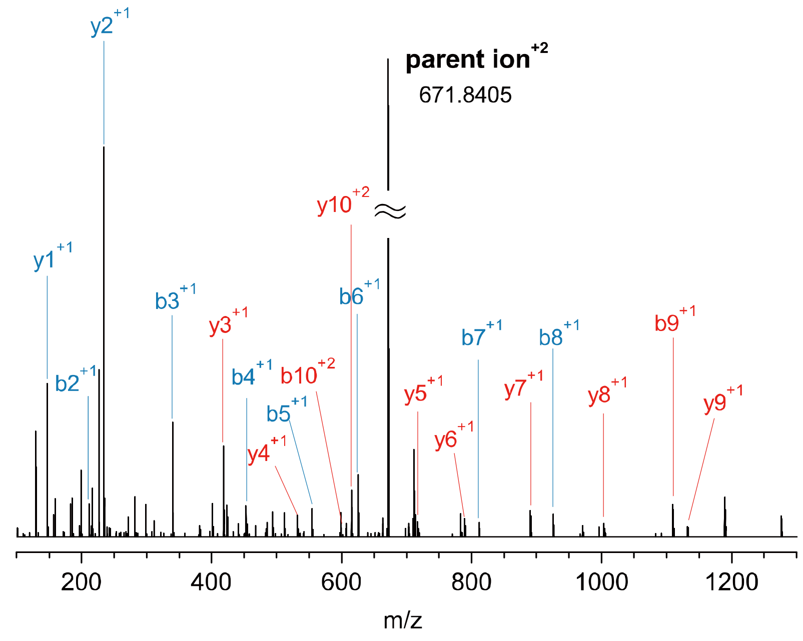

MS/MS data assignment of the key tryptic fragments of dehydrated DarA$_{pk(43-53)}$-**3**.

Ion	Sequence	Obs.	Calc.(unmod.)	Error (ppm)	Shift (ΔDa)	Note
M+2H	IPEITAWNWSK	671.8405 (z = 2)	671.8431 (z =2)	−3.9	2.021	−2×[H]
b2	IP	211.1433	211.1441	−3.8	-	
b3	IPE	340.1853	340.1867	−4.1	-	
b4	IPEI	453.2701	453.2708	−1.5	-	
b5	IPEIT	554.3207	554.3184	+4.1	-	

b6	IPEITA	625.3535	625.3556	−3.5	-	
b7	IPEITAW	811.4297	811.4349	−6.4	-	
b8	IPEITAWN	925.4811	925.4778	+3.6	-	
b9	IPEITAWNW	1109.5392	1109.5415	−2.1	2.0179	−2×[H]
b10	IPEITAWNWS	598.7852(z=2）	598.7904（z=2）	-8.7	2.026	−2×[H]
y1	K	147.1124	147.1128	-2.7	-	
y2	SK	234.1441	234.1448	−3.0	-	
y3	WSK	418.2072	418.2085	−3.1	2.0169	−2×[H]
y4	NWSK	532.2518	532.2514	+0.7	2.0153	−2×[H]
y5	WNWSK	718.3264	718.3307	−6.0	2.02	−2×[H]
y6	AWNWSK	789.3607	789.3678	−9.0	2.0228	−2×[H]
y7	TAWNWSK	890.4153	890.4155	-0.2	2.0159	−2×[H]
y8	ITAWNWSK	1003.4944	1003.4996	−5.2	2.0208	−2×[H]
y9	EITAWNWSK	1132.5339	1132.5422	-7.3	2.0239	−2×[H]
y10	PEITAWNWSK	615.3007 (z=2)	615.3011 (z=2)	-0.6	2.0164	−2×[H]

Supplementary Figure 11. HR-MS/MS analysis of the ^{18}O-incorporated DarA$_{pk(43-53)}$-**2**, showing (A) the shorthand representation and (B) MS/MS spectrum of the ^{18}O-incorporated DarA$_{pk(43-53)}$-**2**.

A

DarA$_{pk(43-53)}$:

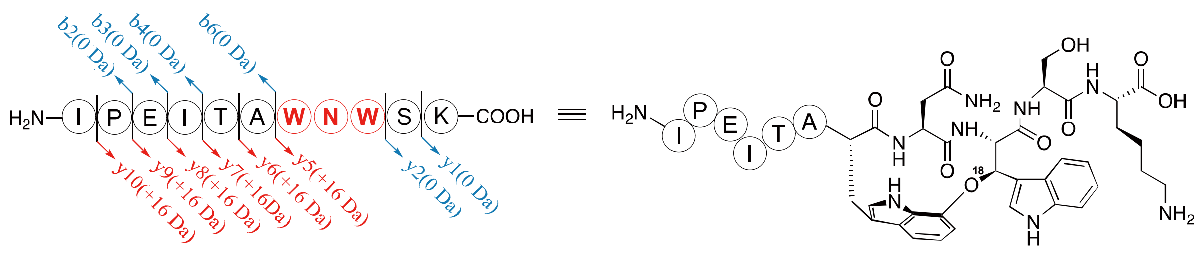

B

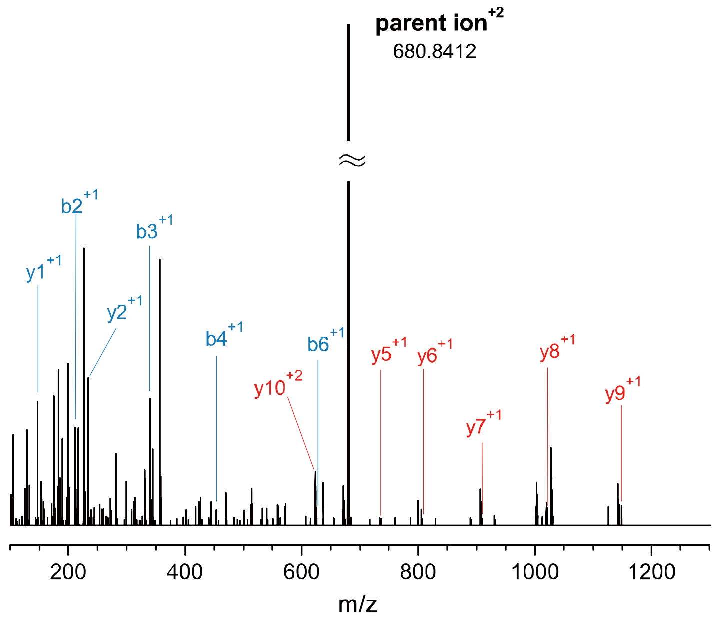

MS/MS data assignment of the key tryptic fragments of the ^{18}O-incorporated DarA$_{pk(43-53)}$-**2**.

Ion	Sequence	Obs.	Calc.(unmod.)	Error (ppm)	Shift (ΔDa)	Note
M+2H	IPEITAWNWSK	680.8412 (z = 2)	680.8427 (z =2)	−1.2	15.9804	$[O]^{18}$–2×[H]
b2	IP	211.1425	211.1441	−7.5	-	
b3	IPE	340.1841	340.1867	−7.6	-	
b4	IPEI	453.2672	453.2708	−7.9	-	
b6	IPEITA	625.3513	625.3556	-6.7	-	
y1	K	147.1118	147.1128	-6.8	-	
y2	SK	234.1432	234.1448	−6.8	-	
y5	WNWSK	736.3279	736.3299	−2.7	15.9715	$[O]^{18}$–2×[H]
y6	AWNWSK	807.3747	807.3670	−9.5	15.9912	$[O]^{18}$–2×[H]

y7	TAWNWSK	908.4080	908.4147	-7.4	15.9768	$[O]^{18}-2\times[H]$
y8	ITAWNWSK	1021.4906	1021.4988	−8.0	15.9836	$[O]^{18}-2\times[H]$
y9	EITAWNWSK	1150.5369	1150.5413	-3.8	15.9791	$[O]^{18}-2\times[H]$
y10	PEITAWNWSK	624.3012 (z=2)	624.3007 (z=2)	+0.8	15.9846	$[O]^{18}-2\times[H]$

Supplementary Figure 12. Detailed comparison of the zoomed-in MSMS spectra for the ^{18}O-labeled vs unlabeled tryptic fragments DarA$_{pk(43-53)}$-**2** generated in ~80% $H_2{}^{18}O$ or in H_2O. The indentical ions (i.e. y1, y2) are shown in blue whereas the ions containing the incorprated O atom (i.e. y5-y8) are shown in red.

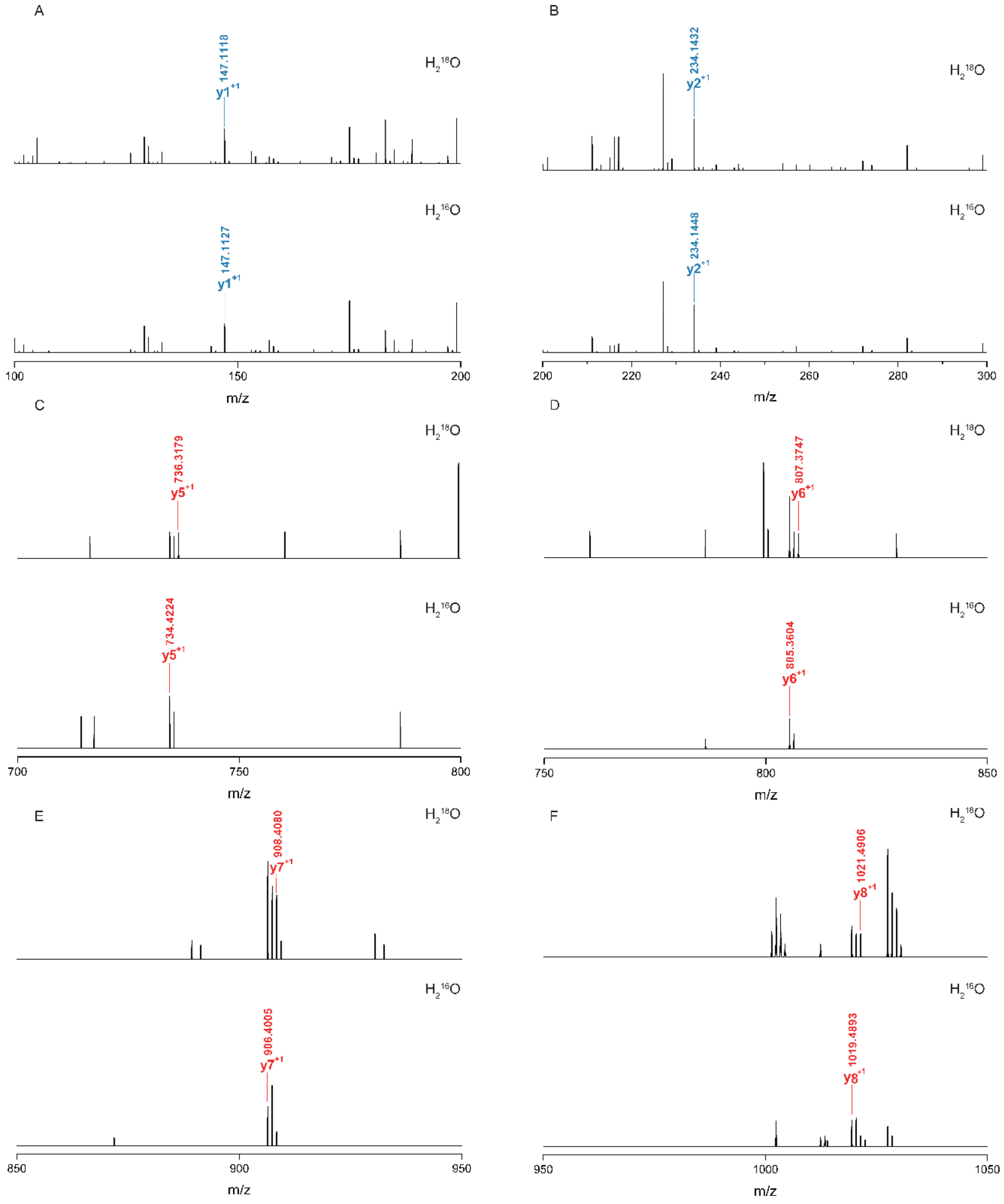

Supplementary Figure 13. HR-MS/MS analysis of DarA$_{yi(55\text{-}66)}$-**1**, showing (A) the shorthand representation and (B) MS/MS spectrum of DarA$_{yi(55\text{-}66)}$-**1**.

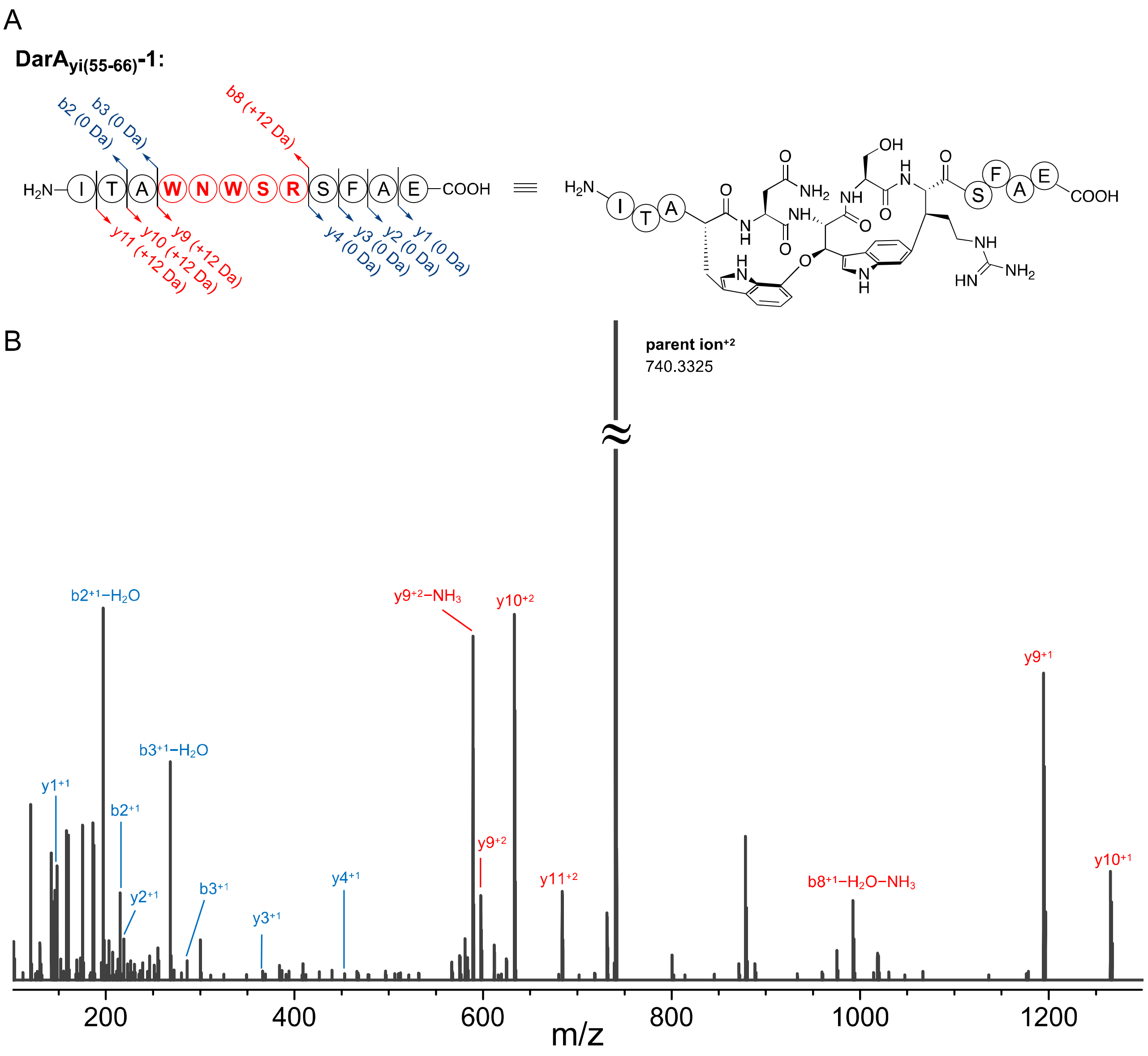

MS/MS data assignment of the key tryptic fragments DarA$_{yi(55\text{-}66)}$-**1**

Ion	Sequence	Obs.	Calc.(unmod.)	Error (ppm)	Shift (ΔDa)	Note
M+2H	ITAWNWSRSFAE	740.3325 (z = 2)	734.3544 (z =2)	−2.5	11.9562	[O]−4×[H]
b2	IT	215.1384	215.1390	−3.2	-	
b2-H_2O	IT	197.1276	197.1285	−4.5	-	
b3	ITA	340.1860	340.1867	−2.0	-	
b3-H_2O	ITA	268.1644	268.1656	−4.5	-	
b8-NH_3-H_2O	ITAWNWSR	992.4373	992.4164	−21.0	11.9427	[O]−4×[H]
y1	E	148.0602	148.0604	−1.3	-	

y2	AE	219.0967	219.0975	−3.6	-	
y3	FAE	366.1596	366.1660	−1.0	-	
y4	SFAE	453.1951	453.1980	−6.4	-	
y9	WNWSRSFAE	1194.4940	1194.4963	−1.9	11.9613	[O]−4×[H]
		597.7496 (z = 2)	597.7518 (z = 2)	−1.8	11.9592	
y9-NH_3	WNWSRSFAE	589.2363 (z = 2)	589.2385 (z = 2)	−1.9	11.9592	[O]−4×[H]
y10	AWNWSRSFAE	1265.5317	1265.5334	−1.3	11.9619	[O]−4×[H]
		633.2670 (z = 2)	633.2703 (z = 2)	−2.6	11.9586	
y11	TAWNWSRSFAE	683.7889 (z = 2)	683.7942 (z =2)	−3.8	11.953	[O]−4×[H]

Supplementary Figure 14. ^{1}H NMR spectrum of DarA$_{yi(55-66)}$-**1**.

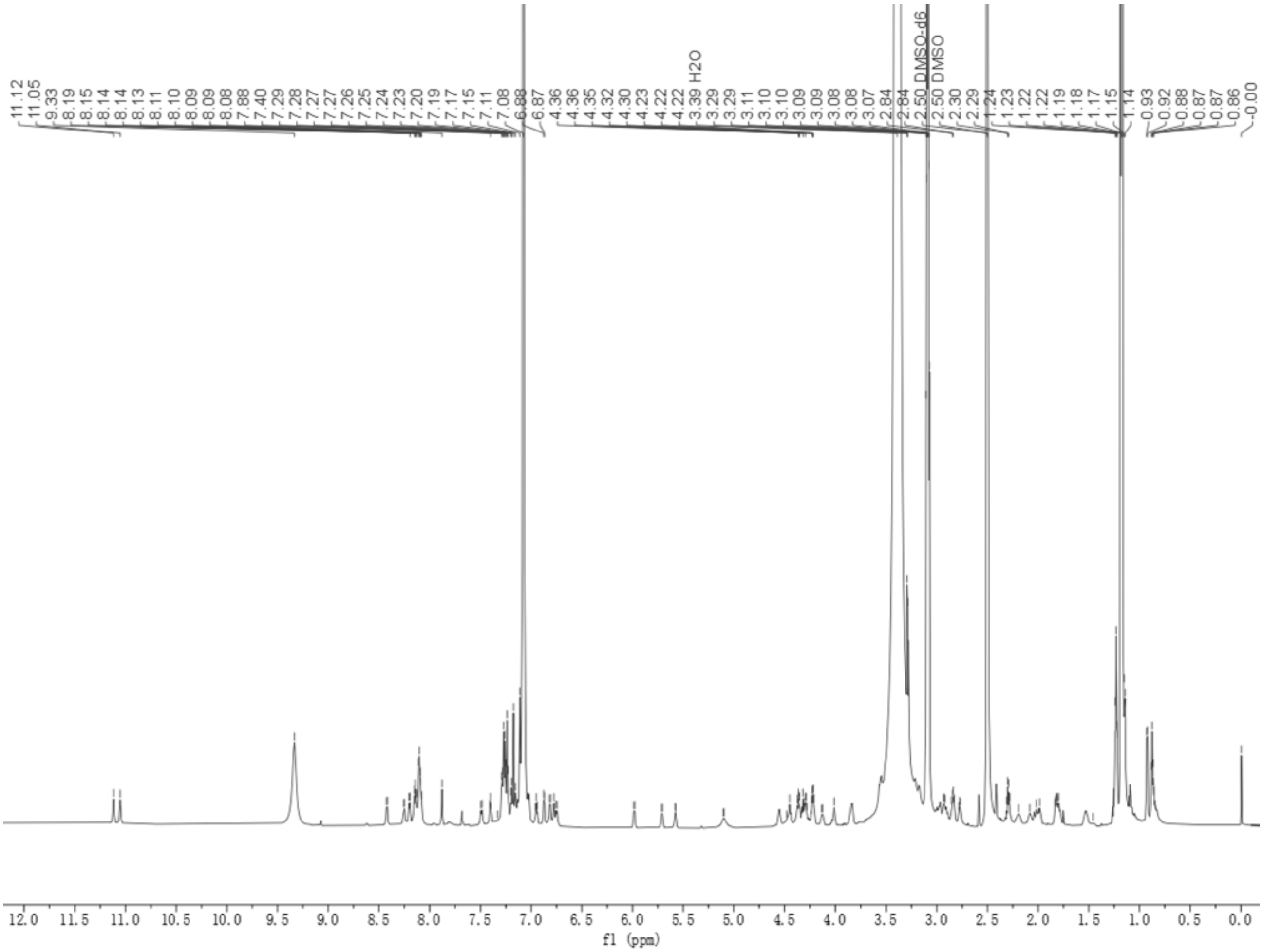

Supplementary Figure 15. ^{1}H-^{1}H COSY spectrum of DarA$_{yi(55\text{-}66)}$-**1**.

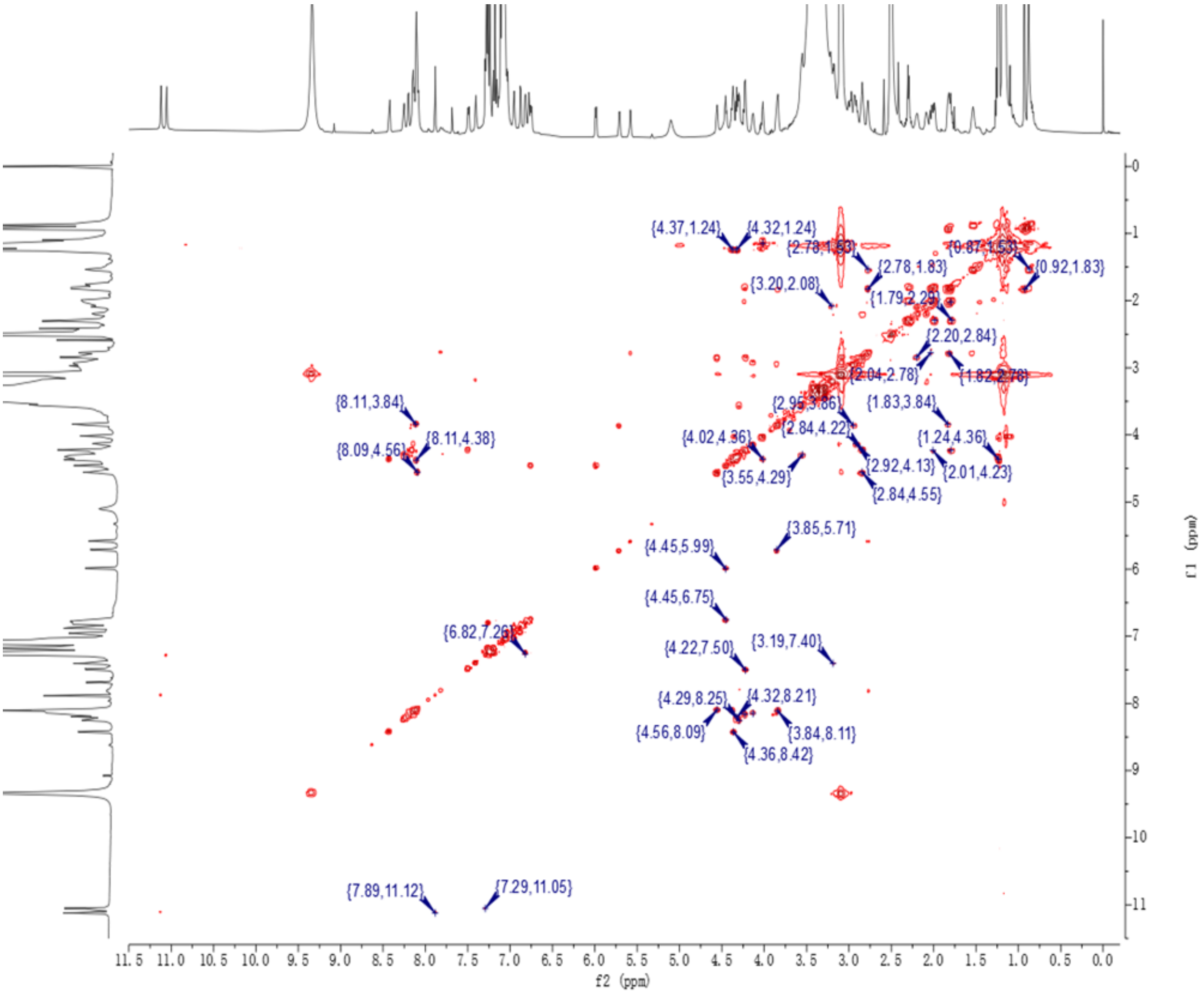

Supplementary Figure 16. HSQC spectrum of DarA$_{yi(55\text{-}66)}$-**1**.

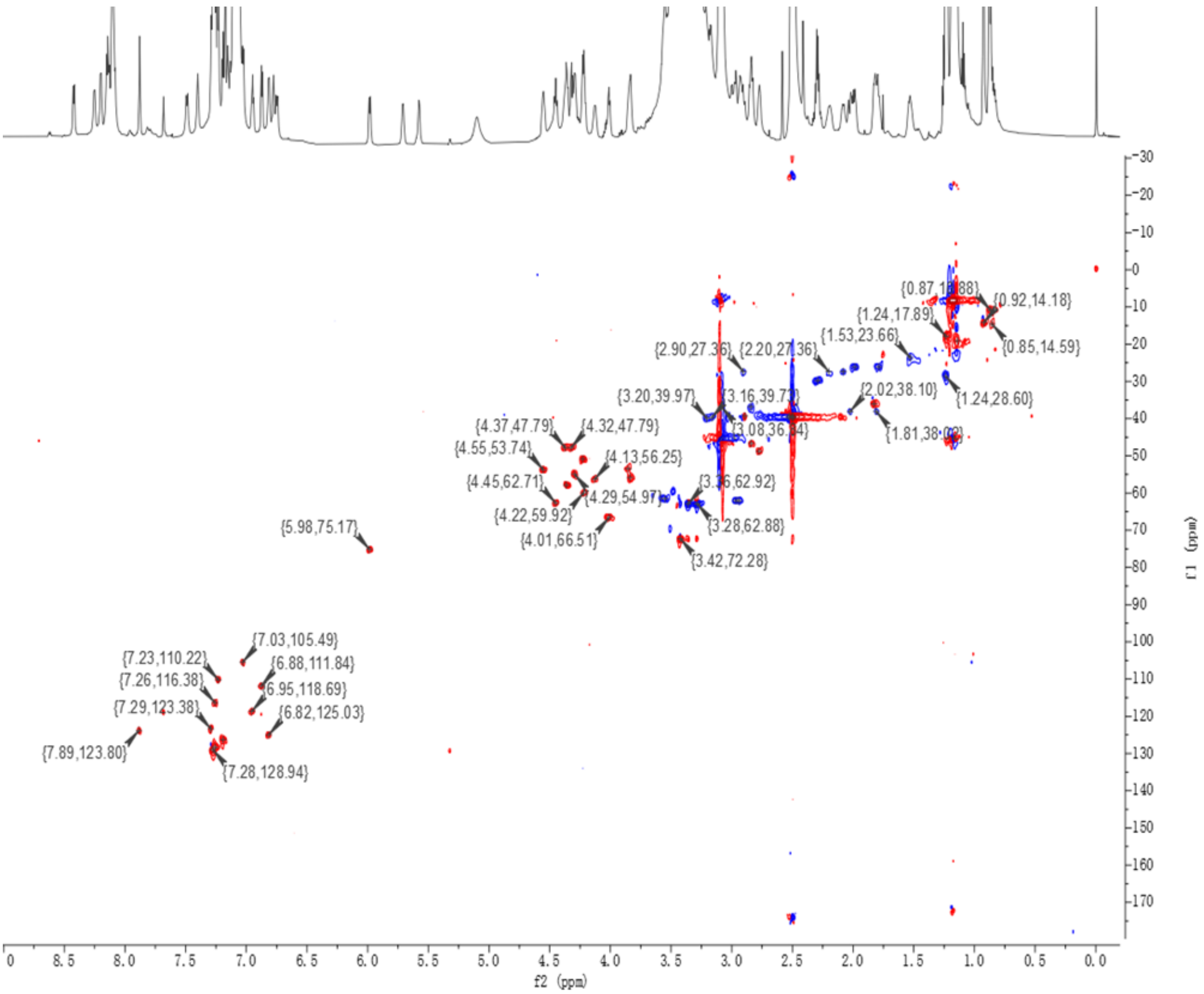

Supplementary Figure 17. NOESY spectrum of DarA$_{yi(55\text{-}66)}$-**1**.

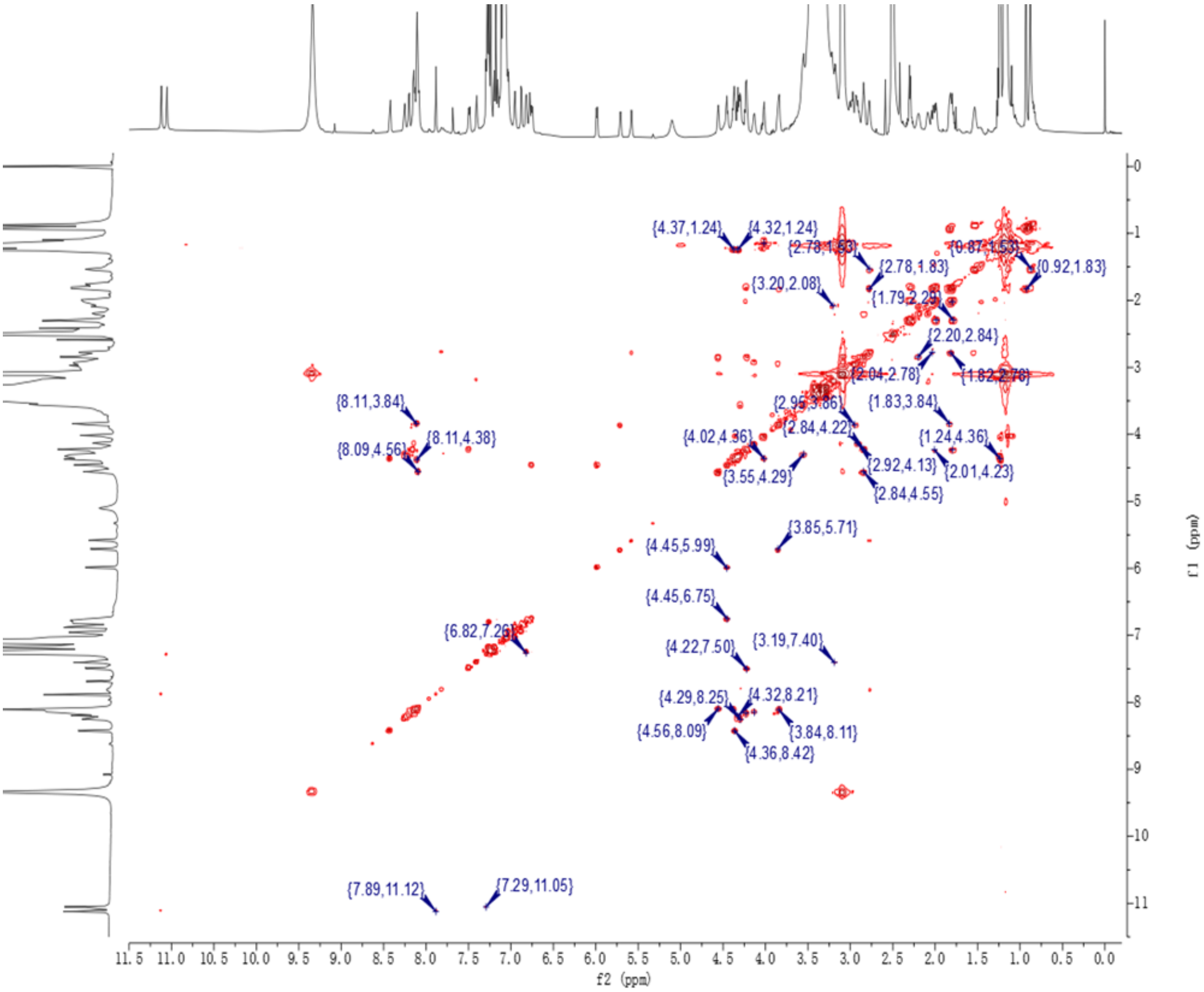

Supplementary Figure 18. Structural validation of DarA$_{yi(55\text{-}66)}$-**1** by combined NMR spectroscopy and DFT GIAO calculation. (A). COSY correlations and planar structure of DarA$_{yi(55\text{-}66)}$-1. (B) Key NOESY correlations and vicinal scalar couplings observed within the bicyclic scaffold. (C) Model substructure of DarA$_{yi(55\text{-}66)}$-**1** and illustrations for the planar chirality within the macrocycle. For the western *O*-indole ether-bridged cyclophane, the structure is oriented with O atom above C_7 and the view down the O-C_7 axis; for the eastern one, the structure is aligned with Cα above C_β and view down the C_β-C_6 axis. (D) Scatter plot of 20 calculated /experimental ^{13}C NMR chemical shifts for the model substructure. Diagonal dashed line: $y = x$. Inset corresponds to error distribution: the centre line (bold red line) indicates the median; box limits indicate upper and lower quartiles; whiskers indicate 1.5×the interquartile range; hollow square indicates the absolute mean; solid circles indicate the maximum negative (positive) error.

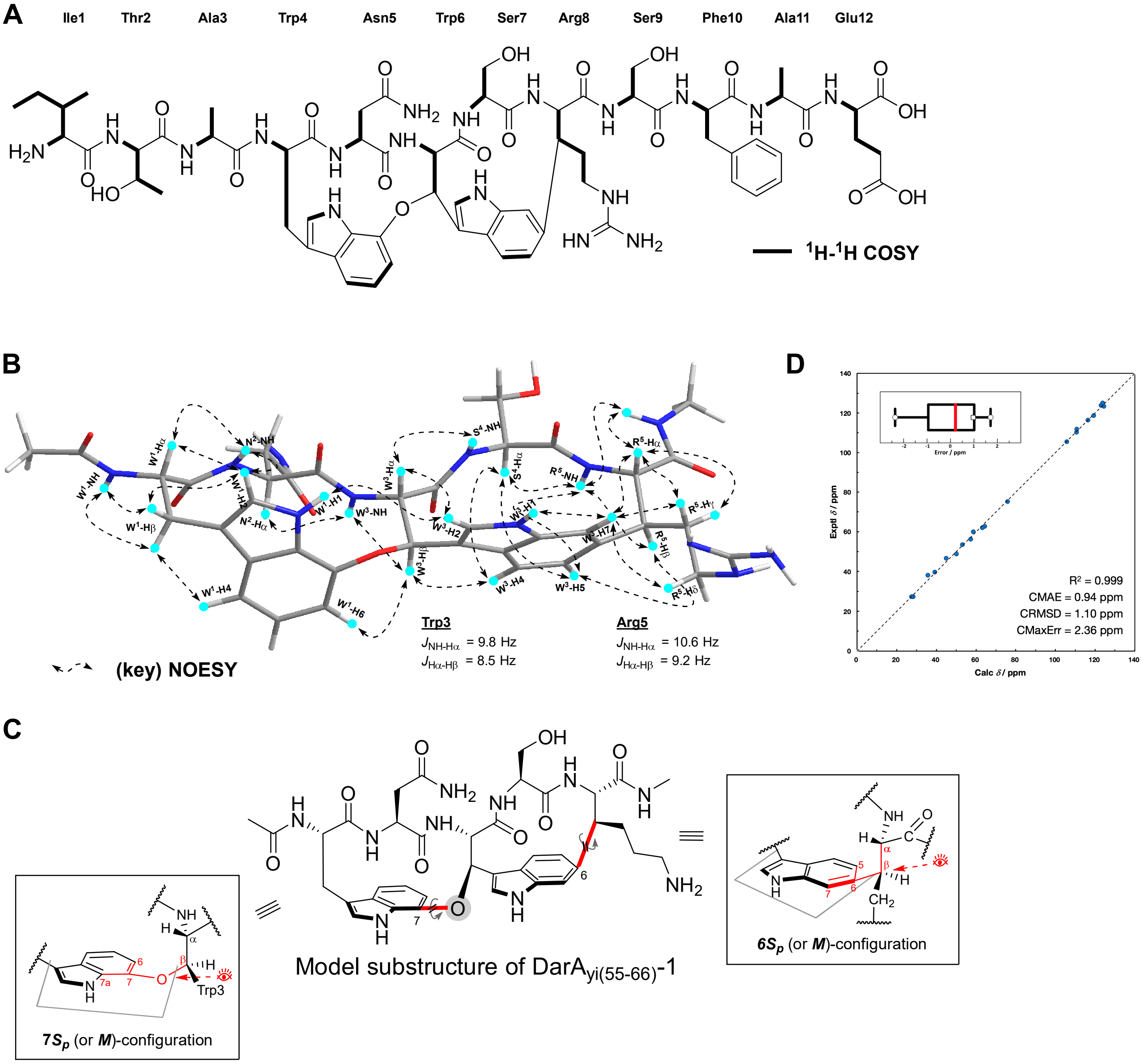

Supplementary Figure 19. HR-MS spectrum of DarA$_{pa}$-**1** modified by DarE$_{pa}$, which contains an ether crosslink between Trp45 and Phe47.

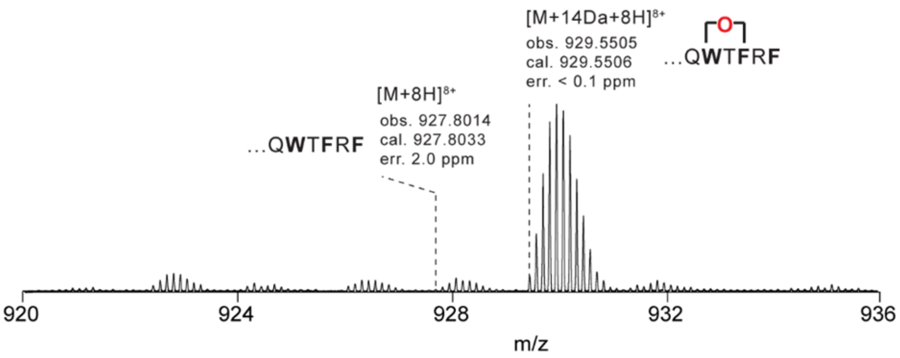

Supplementary Figure 20. Structural analysis of DarA$_{pa(32-49)}$**-1**, showing the EIC (1177.03 ± 0.01), the HR-MS spectrum, and the HR-MS/MS spectrum.

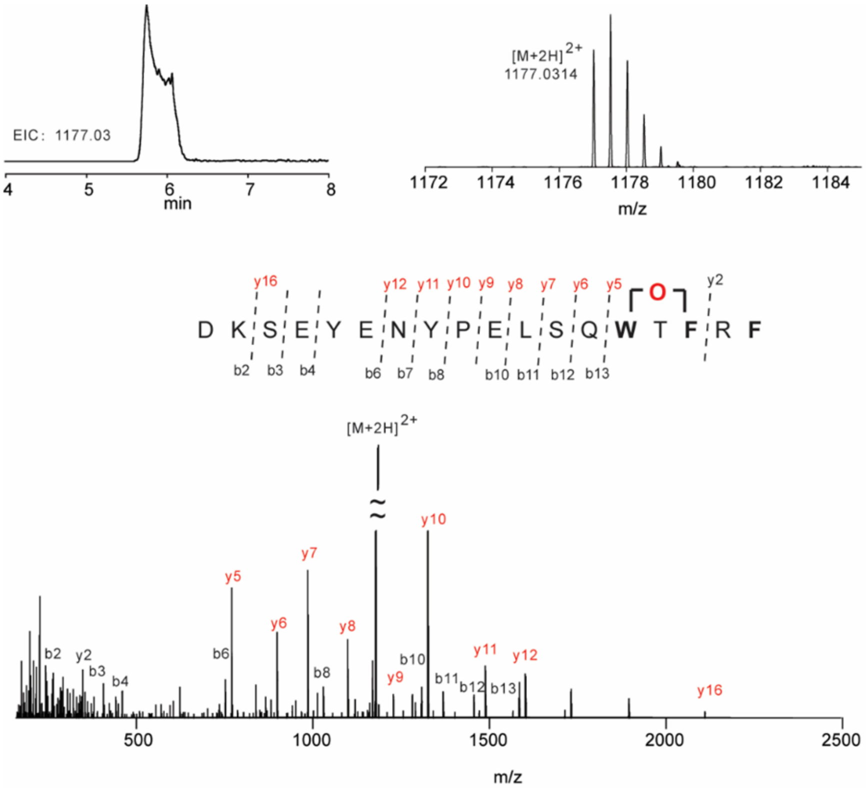

Ion	Calc.	Obs.	Error	Note
[M+2H]2+	1170.0344	1177.0270	-	+14Da
b2	244.1292	244.1302	4.1	
b3	331.1612	331.1600	-3.6	
b4	460.2038	460.2030	-1.7	
b5	623.2671	-	-	
b6	752.3097	752.3107	1.3	
b7	866.3527	866.3539	1.4	
b8	1029.4160	1029.4198	3.7	
b9	1122.4687	-	-	
b10	1255.5113	1255.5133	1.6	
b11	1368.5954	1368.5994	2.9	
b12	1455.6274	1455.6350	5.2	
b13	1583.6860	1583.6826	-2.1	
b14	1769.7653	-	-	crosslink
b15	1870.8130	-	-	crosslink
b16	2017.8814	-	-	
b17	2173.9825	-	-	
y2	322.1874	322.1864	-3.1	
y3	469.2558	-	-	ether crosslink
y4	570.3035	-	-	ether crosslink
y5	756.3828	770.3624	-	+14Da
y6	884.4413	898.4188	-	+14Da
y7	971.4734	985.4523	-	+14Da
y8	1084.5574	1098.5374	-	+14Da
y9	1213.6000	1227.5745	-	+14Da
y10	1310.6528	1324.6329	-	+14Da
y11	1473.7161	1487.6967	-	+14Da
y12	1587.7591	1601.7480	-	+14Da
y13	1716.8016	1730.7775	-	
y14	1879.8650	1893.8275	-	
y15	2008.9076	-	-	
y16	2095.9396	2109.8989	-	+14Da
y17	2224.0346	-	-	

Supplementary Figure 21. HR-MS/MS spectrum of the DarA$_{pa}$-derived monocyclic daropeptide obtained in in vitro proteolysis analysis.

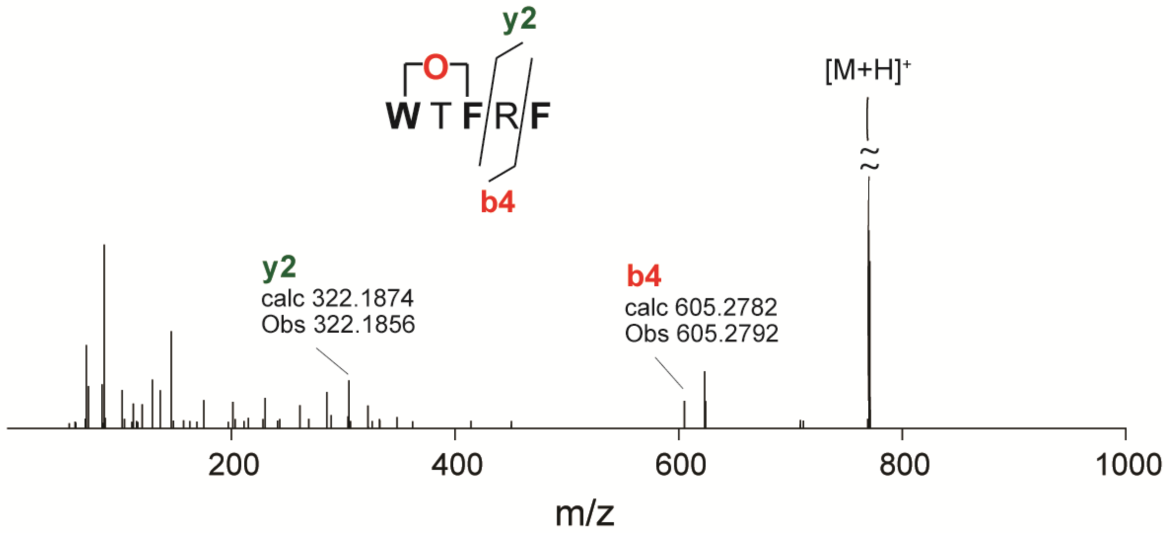

Supplementary Figure 22. LC-HRMS and HR-MS/MS analysis of the tryptic fragement of the DarA$_{pk}$ W51F mutant coexpressed with DarE$_{pk}$. (A) The extracted ion chromatograms (EICs) at m/z = 660.34 ± 0.1 corresponding to the +14 Da modification. (B) The shorthand representation structure and MS/MS spectrum of DarA$_{pk}$(W51F)$_{(43-53)}$ after trypsin digestion.

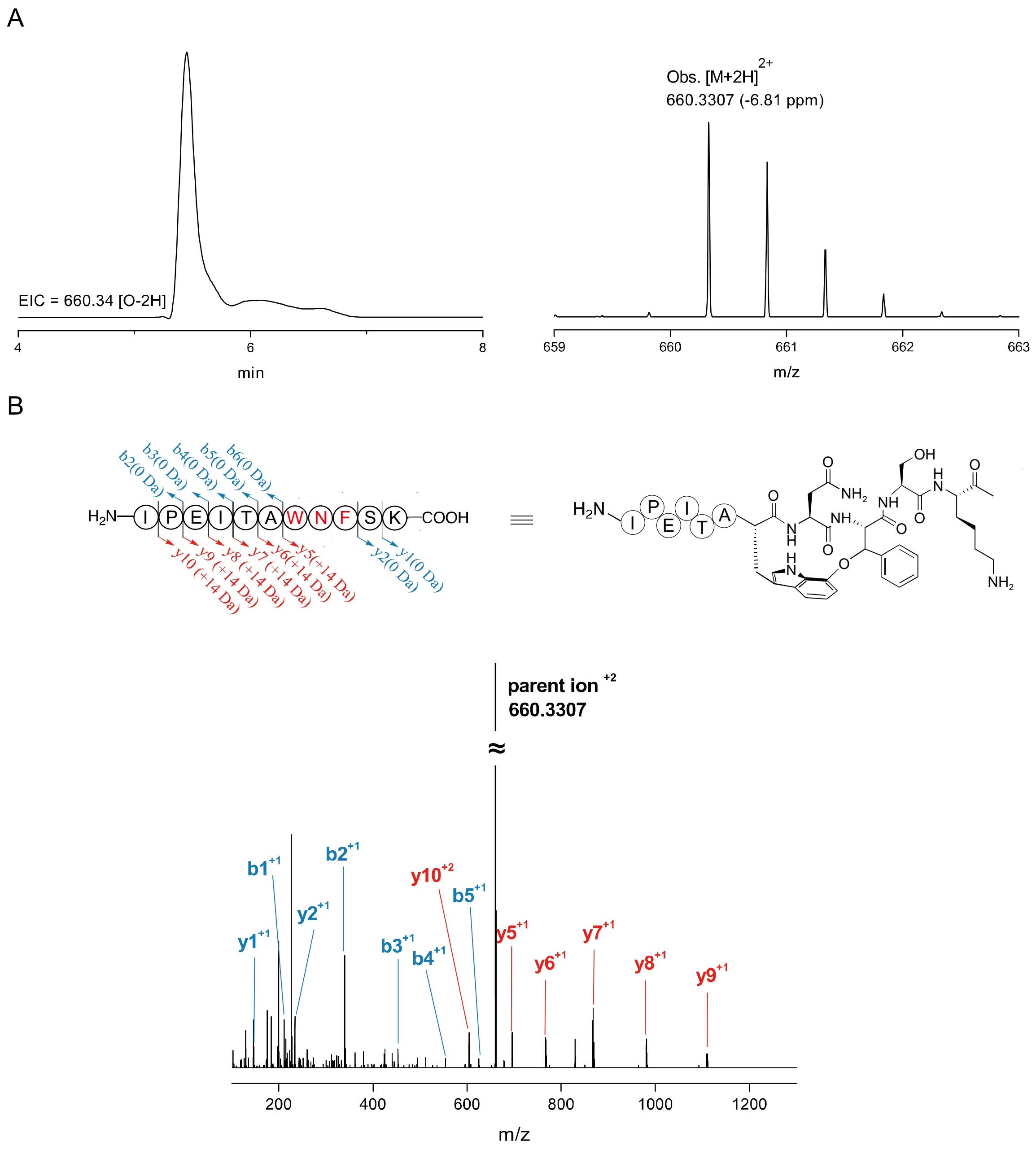

ion	Sequence	Obs.	Calc (unmod.)	Error (ppm)	Shift (ΔDa)	Note
M+2H	IPEITAWNFSK	660.3307 (z = 2)	660.3352 (z =2)	-6.81	13.9704	[O]−2×[H]
b2	IP	211.1429	211.1441	-5.68	-	
b3	IPE	340.1843	340.1867	-7.05	-	
b4	IPEI	453.2689	453.2708	-4.19	-	
b5	IPEIT	554.3170	554.3184	-2.53	-	
b6	IPEITA	625.3536	625.3556	-3.20	-	
y1	K	147.1118	147.1128	-6.80	-	
y2	SK	234.1432	234.1448	-6.83	-	
y5	WNFSK	695.3096	695.3148	-7.48	13.9741	[O]−2×[H]
y6	AWNFSK	766.3448	766.3519	-9.26	13.9722	[O]−2×[H]
y7	TAWNFSK	867.3920	867.3995	-8.65	13.9717	[O]−2×[H]
y8	ITAWNFSK	980.4808	980.4836	-2.86	13.9765	[O]−2×[H]
y9	EITAWNFSK	1109.5211	1109.5262	-4.60	13.9742	[O]−2×[H]
y10	PEITAWNFSK	603.7911(z=2)	596.8035(z=2)	-3.31	13.9752	[O]−2×[H]

Supplementary Figure 23. LC-HRMS and HR-MS/MS analysis of the tryptic fragement of the DarA$_{pk}$ W49F mutant coexpressed with DarE$_{pk}$. (A) The extracted ion chromatograms (EICs) at m/z = 661.34 ± 0.1 corresponding to the +16 Da modification. (B) The shorthand representation structure and MS/MS spectrum of DarA$_{pk}$(W49F) $_{(43\text{-}53)}$ after trypsin digestion.

A

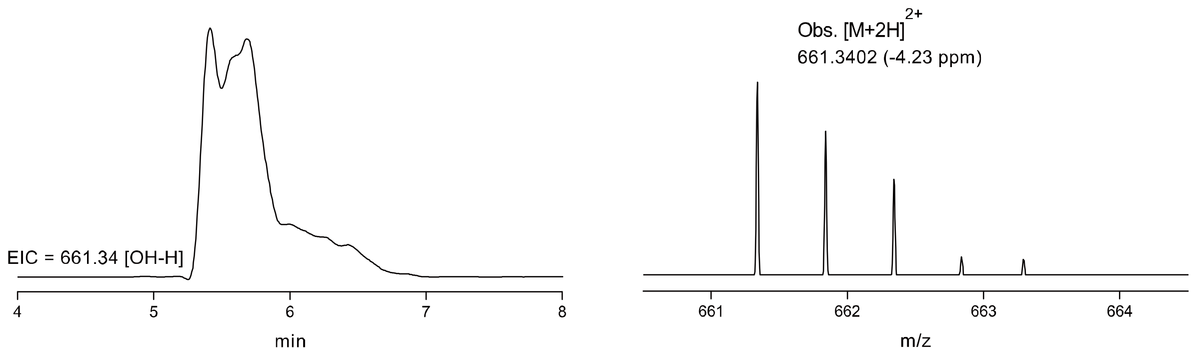

B

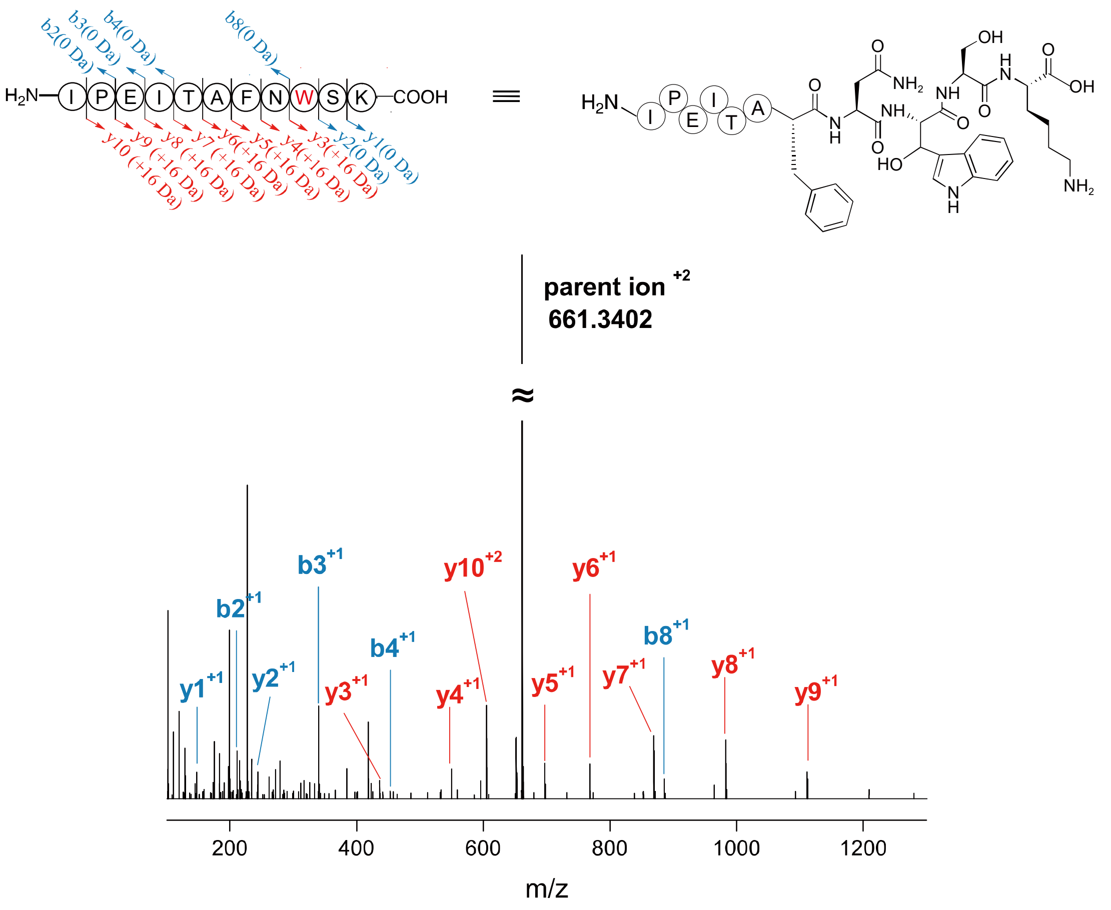

Ion	Sequence	Obs.	Calc (unmod.)	Error (ppm)	Shift (ΔDa)	Note
M+2H	IPEITAFAWSK	661.3402 (z = 2)	653.3455 (z =2)	-4.23	15.9894	[OH]−[H]
b2	IP	211.1422	211.1441	-9.00	-	
b3	IPE	340.1835	340.1867	-9.41	-	
b4	IPEI	453.2706	453.2708	-0.44	-	
b8	IPEITAFN	886.4615	886.4669	-6.09	-	
y1	K	147.1117	147.1128	-7.48	-	
y2	SK	234.1442	234.1448	-2.56	-	
y3	WSK	436.2151	420.2241	-9.17	15.9910	[OH]−[H]
y4	NWSK	550.2539	534.2671	-14.72	15.9868	[OH]−[H]
y5	FNWSK	697.3289	681.3355	-2.15	15.9934	[OH]−[H]
y6	AFNWSK	768.3599	752.3726	-9.89	15.9873	[OH]−[H]
y7	TAFNWSK	869.4108	853.4203	-5.06	15.9905	[OH]−[H]
y8	ITAFNWSK	982.4942	966.5043	-5.19	15.9899	[OH]−[H]
y9	EITAFNWSK	1111.5377	1095.5469	-3.78	15.9908	[OH]−[H]
y10	PEITAFNWSK	604.7975(z=2)	596.8035(z=2)	-5.62	15.9880	[OH]−[H]

Supplementary Figure 24. LC-HRMS and HR-MS/MS analysis of the tryptic fragement of DarA$_{pk.}$co-expression with DarE$_{yi}$. (A) The extracted ion chromatograms (EICs) at m/z = 679.84 ± 0.1 corresponding to the +14 Da modification. (B) The shorthand representation structure and MS/MS spectrum of DarA$_{pk(43\text{-}53)}$ after trypsin digestion.

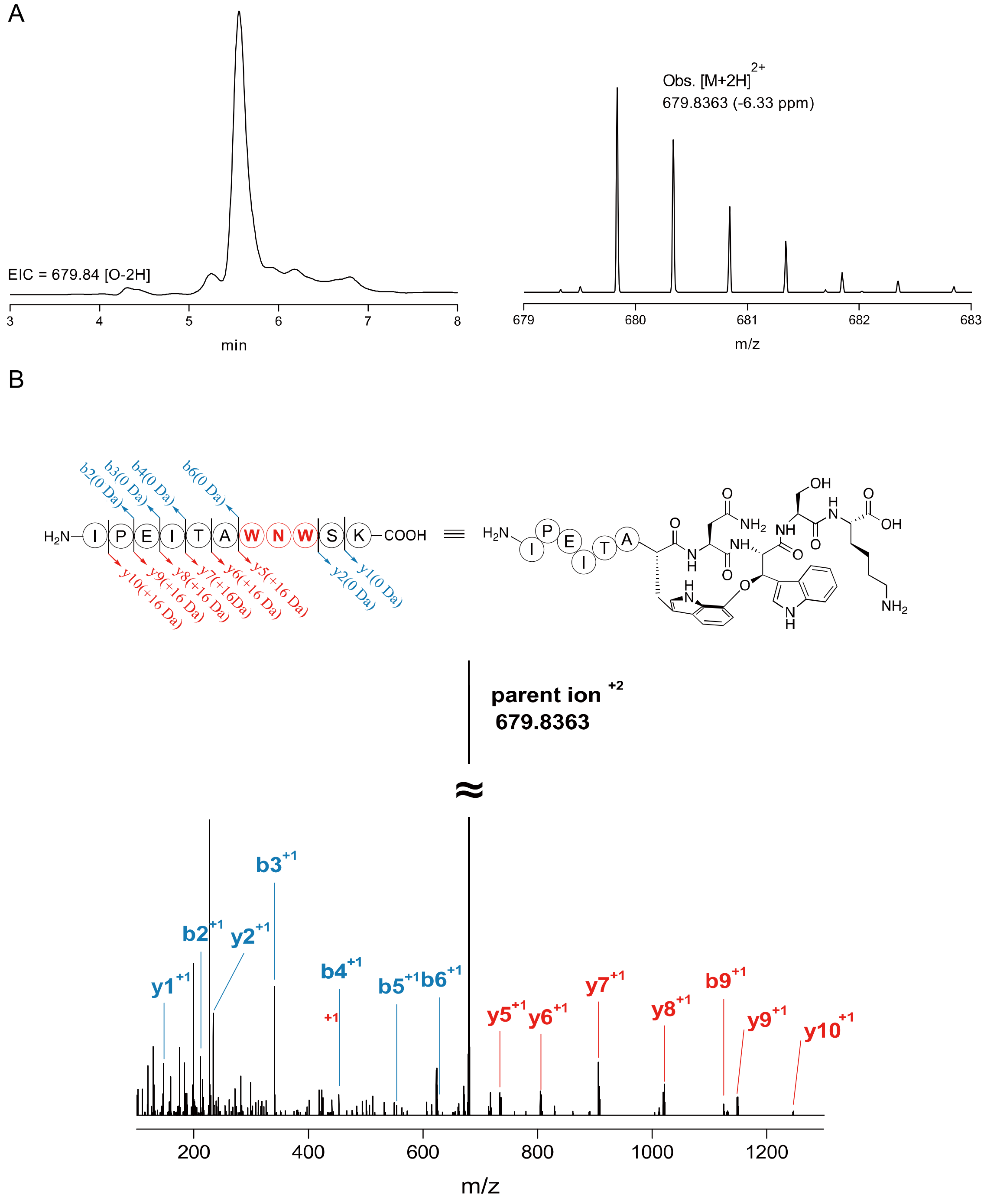

**Ion**	**Sequence**	**Obs.**	**Calc.(unmod.)**	**Error (ppm)**	**Shift (ΔDa)**	**Note**
M+2H	IPEITAWNWSK	679.8363 (z = 2)	672.8510 (z =2)	−6.33	13.9706	[O]−2×[H]
b2	IP	211.1426	211.1441	-7.10	-	
b3	IPE	340.1840	340.1867	-7.94	-	
b4	IPEI	453.2693	453.2708	-3.31	-	
b5	IPEIT	554.3174	554.3184	-1.80	-	
b6	IPEITA	625.3540	625.3556	-2.59	-	
b9	IPEITAWNW	1125.5243	1111.5571	-10.75	13.9672	[O]−2×[H]
y1	K	147.1118	147.1128	-6.80	-	
y2	SK	234.1432	234.1448	-6.83	-	
y5	WNWSK	734.3184	720.3464	-9.94	13.9720	[O]−2×[H]
y6	AWNWSK	805.3531	791.3835	-12.04	13.9696	[O]−2×[H]
y7	TAWNWSK	906.4017	892.4312	-9.6	13.9705	[O]−2×[H]
y8	ITAWNWSK	1019.4847	1005.5152	-9.61	13.9695	[O]−2×[H]
y9	EITAWNWSK	1148.5283	1134.5578	-7.66	13.9705	[O]−2×[H]
y10	PEITAWNWSK	1245.5870	1231.6106	-2.32	13.9764	[O]−2×[H]

Supplementary Figure 25. LC-HRMS and HR-MS/MS analysis of the tryptic fragement of DarA$_{pk}$.co-expression with DarE$_{pa}$. (A) The extracted ion chromatograms (EICs) at m/z = 679.84 ± 0.1 corresponding to the +14 Da modification. (B) The shorthand representation structure and MS/MS spectrum of DarA$_{pk(43-53)}$ after trypsin digestion.

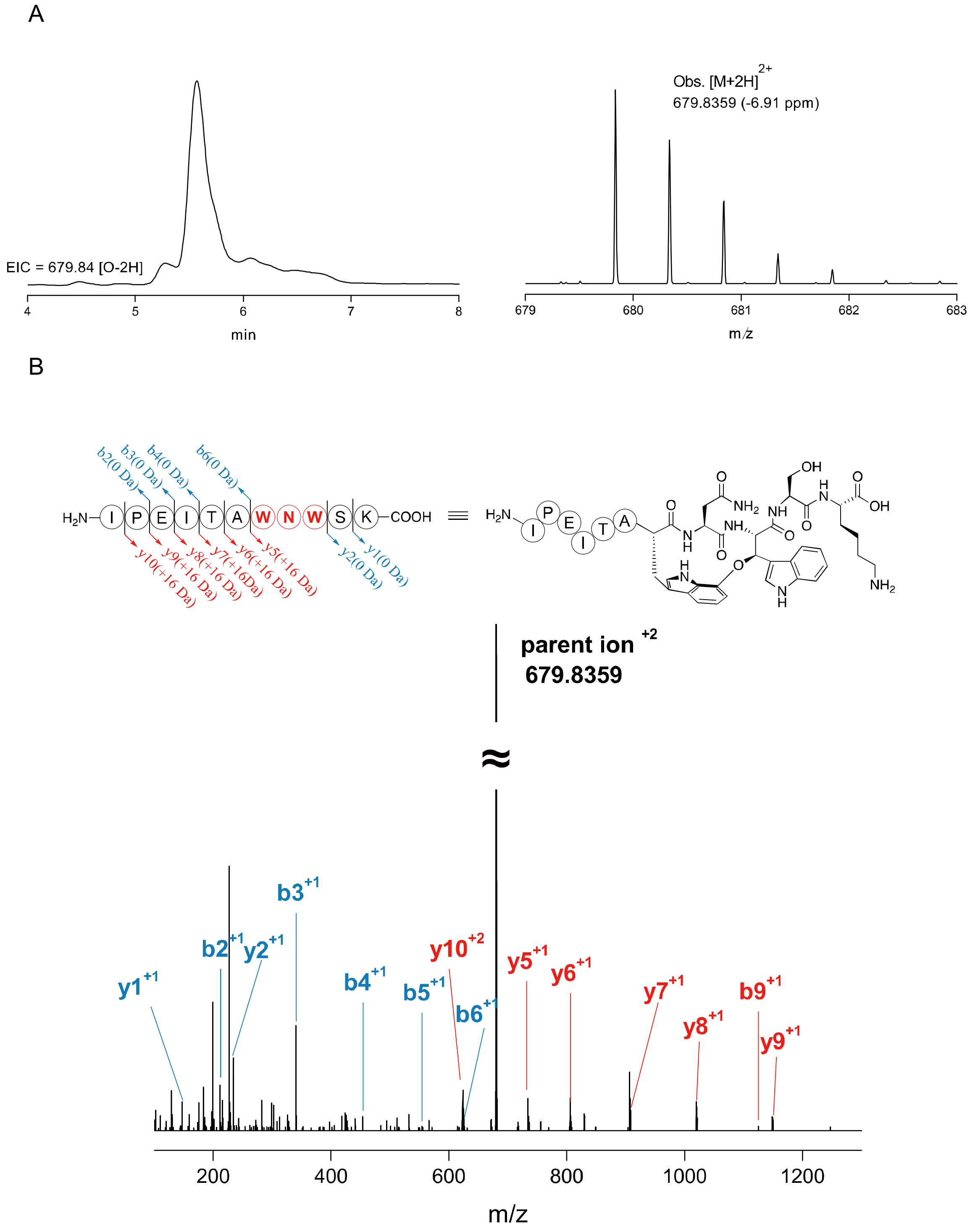

Ion	Sequence	Obs.	Calc.(unmod.)	Error (ppm)	Shift (ΔDa)	Note
M+2H	IPEITAWNWSK	679.8359 (z = 2)	672.8510 (z =2)	−6.33	13.9706	[O]−2×[H]
b2	IP	211.1424	211.1441	-8.05	-	
b3	IPE	340.1838	340.1867	-8.52	-	
b4	IPEI	453.2693	453.2708	-3.31	-	
b5	IPEIT	554.3173	554.3184	-1.98	-	
b6	IPEITA	625.3491	625.3556	-10.39	-	
b9	IPEITAWNW	1125.5154	1111.5571	-18.66	13.9583	[O]−2×[H]
y1	K	147.1119	147.1128	-6.12	-	
y2	SK	234.1433	234.1448	-6.41	-	
y5	WNWSK	734.3291	720.3464	4.63	13.9827	[O]−2×[H]
y6	AWNWSK	805.3567	791.3835	-7.57	13.9732	[O]−2×[H]
y7	TAWNWSK	906.4014	892.4312	-9.93	13.9702	[O]−2×[H]
y8	ITAWNWSK	1019.4862	1005.5152	-8.14	13.9710	[O]−2×[H]
y9	EITAWNWSK	1148.5225	1134.5578	-1.27	13.9647	[O]−2×[H]
y10	PEITAWNWSK	623.2938	623.2986 (z=2)	-7.70	13.9698	[O]−2×[H]

Supplementary Table 1. Complete NMR assignments for DarA$_{yi(55\text{-}66)}$-**1** and darobactin.

Residue	Position	DarA$_{yi(55\text{-}66)}$-**1** (in DMSO-d_6)		darobactin (in H_2O: D_2O 9:1)[8]	
		δ_H (mult., *J* in Hz)	δ_C	δ_H (mult., *J* in Hz)	δ_C
**Ile-1**	NH_2	8.11 (2H, d, 7.4)			
	α	3.84 (1H, overlap)	55.8		
	β	1.83, (1H, overlap)	36.0		
	γ	1.53 (2H, m)	23.7		
	δ	0.87 (3H, t, 7.4)	10.9		
	γ-Me	0.92 (3H, d, 6.8)	14.2		
**Thr-2**	NH	8.42 (1H, d, 8.0)			
	α	4.36 (1H, dd, 8.0, 5.0)	57.9		
	β	4.02 (1H, qd, 6.2, 5.0)	66.6		
	γ	1.14 (3H, d, 6.2)	19.1		
**Ala-3**	NH	8.11 (1H, d, 4.9)			
	C=O		172.2		
	α	4.38 (1H, dq, 7.5, 6.5)	47.8		
	β	1.24 (3H, overlap)	17.9		
**Trp-4**	NH	8.13 (1H, d, 6.5)			
	α	4.13 (1H, br s)	56.3	4.04 (1H, dd, 11.2, 7.7)	57.6
	β	α 2.90 (1H, overlap)<br>β 3.10 (overlap)	27.4	3.30 (1H, dd)<br>3.55 (1H, dd, 14.1,7.6)	29.2
	1	11.05 (1H, s)		10.63 (1H, br s)	
	2	7.29 (1H, s)	123.4	7.35 (1H, br s)	127.6
	3				111
	3a				131.8
	4	6.88 (1H, d, 7.8)	111.8	7.22 (1H, d, 7.7)	116.5
	5	6.95 (1H, t, 7.8)	118.7	7.18 (1H, t, 7.7)	123
	6	7.03 (1H, d, 7.8)	105.5	7.24 (1H, d, 7.7)	111.6
	7				147.9
	7a				131.8
**Asn-5**	NH	5.58 (1H, br d, 5.2)		6.92 (1H, d, 8.1)	
	α	2.78 (1H, br s)	48.8	3.33 (1H, m)	53.7
	β	1.81 (1H, overlap)<br>2.02 (1H, br d, 13.8)	38.1	2.13 (1H, dd, 13.9, 7.2)<br>2.19 (1H, dd, 13.9, 7.2)	41.9
	NH_2	6.78 (1H, overlap)<br>7.16 (1h, overlap)		6.64 (1H, br s)<br>7.31 (1H, br s)	
**Trp-6**	NH	6.75 (1H, d, 9.8)		7.83 (1H, d, 9.8)	
	α	4.45 (1H, dd, 9.8, 8.5)	62.7	4.69 (1H, dd, 10.2, 9.1)	66.1
	β	5.99 (1H, d, 8.5)	75.2	6.18 (1H, d, 9.1)	79.5
	1	11.12 (1H, s)		10.44 (1H, br s)	
	2	7.88 (1H, s)	123.8	7.85 (1H, br s)	
	3			7.45 (1H, d)	114.5

	3a				127.8
	4	7.26 (1H, d, 8.0)	116.4	7.45 (1H, d)	120
	5	6.82 (1H, d, 8.0)	125.0	6.96 (1H, d)	127.7
	6				135.7
	7	7.23 (1H, br s)	110.2	7.48 (1H, br s)	113.3
	7a				139.9
**Ser-7**	NH	5.71 (1H, br d, 7.5)		6.95 (1H, d)	
	α	3.85 (1H, overlap)	53.5	3.95 (1H, m)	56.9
	β	2.94 (1H, m)<br>2.96 (1H, m)	62.1	3.14 (1H, dd)<br>3.22 (1H, dd)	64.8
**Arg-8**	NH	7.49 (1H, d, 10.6)		7.88 (1H, d, 10.7)	
	α	4.22 (1H, dd, 10.6, 9.2)	59.9	4.25 (1H, t, 10.9)	63
	β	2.84 (1H, overlap)	46.6	3.03 (1H, m)	51
	γ	2.08 (1H, overlap)<br>2.18 (1H, overlap)	27.4	2.08 (1H, m)	28.5
	δ	3.16 (1H, overlap)<br>3.20 (1H, overlap)	39.7	2.99 (1H, m)	42.4
**Ser-9**	NH	8.25 (1H, d, 7.2)		8.62 (1H, d, 7.3)	
	α	4.29 (1H, m)	55.0	4.46 (1H, m)	58.5
	β	3.55 (2H, m)	61.5	3.8 (1H, d, 5.5)	64
**Phe-10**	NH	8.09, (1H, d, 8.2)		8.14 (1H, d, 7.4)	
	α	4.55 (1H, td, 8.2, 4.2)	53.7	4.64 (1H, dt, 7.7, 5.9)	57.9
	β	2.84 (1H, overlap)<br>3.08 (1H, overlap)	36.8	3.11 (1H, dd)<br>3.22 (1H, dd)	39.8
	1				139.6
	2/4	7.27 (2H, overlap)	128.3	7.32 (1H, d)	132.3
	3/5	7.28 (2H, overlap)	128.9	7.42 (1H, t, 7.49)	131.5
	6	7.20 (1H, t, 7.1)	126.3	7.37 (1H, t, 7.08)	129.9
**Ala-11**	NH	8.2 (1H, d, 7.5)			
	C=O		172.2		
	α	4.32 (1H, dq, 7.5, 6.5)	47.9		
	β	1.24 (3H, overlap)	17.9		
**Glu-12**	NH	8.15 (1H, d, 8.1)			
	α	4.23 (1H, td, 8.1, 5.5)	50.9		
	β	1.79 (1H, overlap)<br>1.99 (1H, m)	26.1		
	γ	2.29 (1H, m)	29.8		

Supplementary Table 2. The encoding sequence of the recombinant plasmids in this study.

**His6-DarA$_{pk}$**	MGSSHHHHHHSQDPMHNTLNETVKTQEALNSLAASFKETELSITDKALNELSNKPKIPEITAWNWSKSFQEI*
**His6-DarE$_{pk}$**	MGSSHHHHHHSQDPMDTIIPIKYLDSDESSILKKSSKINYRQLACRIIGEISAEKILDDDELALYNKEISIHFSPEIINANKLVVVVKATRLCNLRATYCHSWAEGKGNTLTFFNLMRSIHRFLSLPNIKRFEFVWHGGEVTLLNVNYFKKLIWLQEQFKKPDQVITNSVQTNAVNIPEDWLVFLKGIGMGVGISVDGIPEIHDSRRLDYRGRPTSHKVAASMKKLRSYGIPYGALIVVDRDVYESNIEKMLSYFYEIGLTDIEFLNIVPDNRCQPGDDPGGSYITYHNYINFLSKVFRVWWNGYQGKINIRLFDGFIDSIKSSQKKMSDCYWAGNCSQEIITLEPNGTVSACDKYVGAEGNNYGSIIDNDLGNLLSKSNTNKDHLKEEMESYEKMHQCKWFHLCNGGCPHDRVTNRKHNPNYDGSCCGTGGLLETIKQTIAA*
**Chemically synthesized DarA$_{pk}$**	HNTLNETVKTQEALNSLAASFKETELSITDKALNELSNKPKIPEITAWNWSKSFQEI
**DarB$_{pk}$**	MGNAINISESMKRFNILVSILSVSIAILAIYLLSTAFYSDARVDNYYQDNGNIYRVETTFNLPNGEKVRSAKSPLPLIDELEKDKRINRADYFFKLNTTIELQGKKITKVPVFAVSHQFLNTLSPFKKIDNILGANEIYITKEFNNRYLGLENPKGKSIVLDNETYIIKDIVEKRHDSSLNMNAIISFKPTLIKNYNKEIFNWYDTHAYIFIKLSDEKSISNRDALLNHIIATKAPNLPGAPFTASEFIHLSLKKSKDIHYQDGLPDEISIGLSKEIIYSSYIAFAFILFSTLTNYYNLTSADSAEKRDIYQLKKALGASTFNIICDSLPAMLIKIILSTLIFLLLIITIFNVSLYINNIFTLLEADSLIYLGIFSVVIAFIFISSIHFSFSLRFYIYQRNRKMDMRYESTLASWFRKSSLAIQLLVSGISIYLVTGLTNQYIHLVDLHTPYDNAKTISIAINNDNNKKSTIDELSYEFIRKYNTSKITLSNWRPFDMSRENITINYSGQQNRNNYISSNVITADENFTDVWRMKILAGGDKRVYQSDNADVVHALVTKEFLKQNGILKYDDIFNNYYWYTMEDKKIQIRFIQIIDDFNLGAVDEPFRPIVVFIKKDHGKYASLNLNNMKNLSPVLNELSKQGFDNNEIDLTSHLFNSYYDNYLKVIKLSSAVAIFSVLLLVIACFTTSMTDFNAMKRELSIMESIGGSIYTNLIHFLSKSVIPIIASIFVAYISGRTILVYLLDEYSVIYTDSFVYSAIALSATMIFTVIIMMTTYLVNYRKLSATKFL*
**DarC$_{pk}$**	MDIEIRKKGIHNREKLLILIFFIAFISFVVFIIYYVVSAKDAFVNENEVTQFQVSSKVDSDILKTRAVVVPDKSVSISTEIGGIVTDIMKKPSQDVLKNEEIVKLSNFNFTLNNSSMLADVTDKLNNLINIRINLQSDYRDINNRFLEAAKELKEVEDKLKRYHDLVHKNYISKETMSDLRIKRDYWHDVYLFYKKLKNDKDRDISKQLKEIDEFVEKQRKLSDIIENGFEQLSIKSPIAGNISSLDLILGQRL

	KPGDKIAIVDDLSNFYFESEINEYYLNKITYHSSASLIYNNGKIPLLVKLIS SEVNNGTFKVRFELEDKKTINFKRGQSVDVMIKLDEDRKIFSVPSSMVFS IDNKNYVFVYHPDKKIARRIQVSTGQDNGSDIEIKSGVTEGQTLVSFGKN KLVNNDTVRIE*
**DarD$_{pk}$**	MISMMNICKSYKTKFIQTNVLNDINLNIDKGEFISIMGASGSGKSTLLNVI GMFETIDSGQLTLNNSNILTMKYSEKISFRREFIGYIFQSFNLLPNLTVFEN IELPLKYRGFPKKQRKSKVFDAINSFGLENRENHKPIQLSGGQQQRVAIA RAMIANPTILLADEPTGNLDSVNGQNILSSLKELNENGTTIVMVTHSVEA AAFSDRILTMRDGHLLV*
**His$_6$-DarA$_{yi}$**	MGSSHHHHHHSQDPMNISKQSDDKRTKHSHLMALKGKLQSLEESFKNN ALYINDNEIEKIKNNTLRSKITAWNWSRSFAE*
**DarE$_{yi}$**	MRDIIPIVNIDSVNIRDINIRKLEELTSHEYKALSAYLIGKIPASQLLDGDEL TLFNKELTHHMEVKAINADKLVIVLKATRLCNLRCTYCHSWAEGPNQSI SFDILIRAIHKILSIPNVNRFEFVWHGGEVTLLKPLFFKKLIWLQQQFKRPE QYITNTMQSNIVNLSEEWLTFIKGIGMNVGISLDGVPAVNDKRRVDYRG KGTSERVAMGIKKLQHYNILYGALIVVDREVYQTDMREMLDYFISIDLT GIEFLNIVPDNRLAAGEYIGDNFISYAEFIKFLSDLFIIWIKDYREKIHITIFE DFIKALKYPDEKLSACYWSGNCSQEIITLEPNGDISPCDKYRGDANSIYGS ILNIELADLLANSSHNQQAVIEEIEATKKMHHCKWFSICHGGCPHDRVIN RRHTKGYNDTCCGTGKLLATIEDYLATTG*
**His$_6$-DarA$_{pa}$**	MGSSHHHHHHSQDPMNVSTKNHDAKLFPQGDNSKNRDNRETNNNRDK SEYENYPELSQWTFRF*
**DarE$_{pa}$**	MSEKIEVINIDENSDMSTNYTKKRPKIPQELYAYEDIKKIPIQDILSDEEIEI YENEMQKKIEVKPLGSNKLVVVTKATRLCNLRCSYCHSWAEGKNNTMS FSSLVAMTKRILDIPDLIRIEFVWHGGEVTRLRPEFFMKFIWLQQHIKRDN QFVTNAIQTNAVNISEKWIELIKRLNISVGISLDGHPEINDSRRVDLKGIGT SKRIEKTIQTFRNNNIKYGGLVVVDRLILESNKRELLDYFARIHLHDLVFL DIVPDNRLLPGESPGDSYINYFEYMDFLSELFLIWWKEYKDTINIPLFNSF VGVLKGASNVLMSCYWSEKCHQEVVTIEPNATVSACDKYVGSENFYYG SLLDNDLKTLLENSIHYEKSEKEEIDNNNNMKNCQWFNLCHGGCPQTRI SNRKHNKMYSDTLACCGTGDKLLNTISGVL*

Supplementary Table 3. Plasmids used in this study.

**Plasmid**	**Purpose**	**Source**
E. coli DH5α	Host strain for cloning	TAKARA
E. coli BL21(DE3)	Host strain for expression	TAKARA
E. coli BL21star (DE3)	Host strain for expression	TAKARA
pET-28a(+)	Kan^R, Expression vector	Novagen
pRSFDuet	Kan^R, Expression vector	Novagen
pETDuet	Kan^R, Expression vector	Novagen
$pRSFDuet\text{-}darA_{pk}E_{pk}$	Kan^R, Co-expression of $DarA_{pk}E_{pk}$	This study
$pETDuet\text{-}darBCD_{pk}$	Amp^R, Co-expression of $DarBCD_{pk}$	This study
$pRSFDuet\text{-}darA_{pk}E_{pk(C83A)}$	Kan^R, Co-expression of $DarA_{pk}$/$DarE_{pk}$-C83A	This study
$pRSFDuet\text{-}darA_{pk(K53A)}E_{pk}$	Kan^R, Co-expression of $DarA_{pk}$-K53A /$DarE_{pk}$	This study
$pRSFDuet\text{-}darA_{pk(W51F)}E_{pk}$	Kan^R, Co-expression of $DarA_{pk}$-W51F /$DarE_{pk}$	This study
$pRSFDuet\text{-}darA_{pk(W49F)}E_{pk}$	Kan^R, Co-expression of $DarA_{pk}$-W49F /$DarE_{pk}$	This study
$pET28a\text{-}darA_{pk}$	Kan^R, Expression of $DarA_{pk}$	This study
$pRSFDuet\text{-}darEA_{pk}$	Kan^R, Expression of $DarE_{pk}$	This study
$pRSFDuet\text{-}darAE_{yi}$	Kan^R, Co-expression of $DarAE_{yi}$	This study
$pRSFDuet\text{-}darAE_{pa}$	Kan^R, Co-expression of $DarAE_{pa}$	This study
$pRSFDuet\text{-}darA_{pk}E_{yi}$	Kan^R, Co-expression of $DarA_{pk}E_{yi}$	This study
$pRSFDuet\text{-}darA_{pk}E_{pa}$	Kan^R, Co-expression of $DarA_{pk}E_{pa}$	This study

Supplementary Table 4. Primers used in this study.

Primer	Oligonucleotide sequence (5' to 3')
$DarA_{pk}$_For	GGTCGCGGATCCGAATTCATGCACAACACCCTGAAC
$DarA_{pk}$_Rev	GAGTGCGGCCGCAAGCTTTTAAATCTCTTGGAAGCTCTTGC
$DarEA_{pk}$-*BamH*I_For	CGCGGATCCGATGGACACCATCATTCCGATCAAG
$DarEA_{pk}$-*Hind*III_Rev	CCCAAGCTTTTACGCCGCAATGGTTTGTTTGATG
$DarEA_{pk}$-*Nde*I_For	CAGCAGCGGTTTCTTTACCAGACTCGAGTTAAATCTCTTGGAAGCTCTTG
$DarEA_{pk}$-*Xho*I_Rev	CTTCCAAGGAGGTGGCAGCCAGGATCCGATGCACAACACCCTGAACGAGACCG
$DarAE_{pk}$-C83A_For	AACCTGCGTGCCACCTACTGCCACAGCTGGGCGGAGGG
$DarAE_{pk}$-C83A_Rev	CAATAGGTAGCACGAAGGTTGCATAATCGTGTGGCCTTC

Supplementary References

1. Gerlt JA, Bouvier JT, Davidson DB, Imker HJ, Sadkhin B, Slater DR, *et al.* Enzyme Function Initiative-Enzyme Similarity Tool (EFI-EST): A web tool for generating protein sequence similarity networks. *Biochim Biophys Acta* 2015, **1854**(8): 1019-1037.

2. Zallot R, Oberg N, Gerlt JA. The EFI Web Resource for Genomic Enzymology Tools: Leveraging Protein, Genome, and Metagenome Databases to Discover Novel Enzymes and Metabolic Pathways. *Biochemistry* 2019, **58**(41): 4169-4182.

3. Cline MS, Smoot M, Cerami E, Kuchinsky A, Landys N, Workman C, *et al.* Integration of biological networks and gene expression data using Cytoscape. *Nat Protoc* 2007, **2**(10): 2366-2382.

4. Ma S, Chen H, Li H, Ji X, Deng Z, Ding W, *et al.* Post-Translational Formation of Aminomalonate by a Promiscuous Peptide-Modifying Radical SAM Enzyme. *Angew Chem Int Ed* 2021, **60**(36): 19957-19964.

5. Lanz ND, Grove TL, Gogonea CB, Lee KH, Krebs C, Booker SJ. RlmN and AtsB as models for the overproduction and characterization of radical SAM proteins. *Methods Enzymol* 2012, **516**: 125-152.

6. Lodewyk MW, Siebert MR, Tantillo DJ. Computational prediction of 1H and 13C chemical shifts: a useful tool for natural product, mechanistic, and synthetic organic chemistry. *Chem Rev* 2012, **112**(3): 1839-1862.

7. Kutateladze AG, Reddy DS. High-Throughput in Silico Structure Validation and Revision of Halogenated Natural Products Is Enabled by Parametric Corrections to DFT-Computed (13)C NMR Chemical Shifts and Spin-Spin Coupling Constants. *J Org Chem* 2017, **82**(7): 3368-3381.

8. Imai Y, Meyer KJ, Iinishi A, Favre-Godal Q, Green R, Manuse S, *et al.* A new antibiotic selectively kills Gram-negative pathogens. *Nature* 2019, **576**(7787): 459-464.
